# A novel role of tRNA-derived fragments in porcine granulosa-oocyte cell communication and cuproptosis

**DOI:** 10.1371/journal.pgen.1012119

**Published:** 2026-04-30

**Authors:** Linyuan Shen, Xue Zhao, Shuang Wu, Yuhang Lei, Shuang Liang, Saihao Wang, Haodong Dai, Yan Wang, Lei Chen, Ye Zhao, Mailin Gan, Shijun Xiao, Guangbin Zhou, Li Zhu

**Affiliations:** 1 Farm Animal Germplasm Resources and Biotech Breeding Key Laboratory of Sichuan Province, Sichuan Agricultural University, Chengdu, China; 2 State Key Laboratory of Swine and Poultry Breeding Industry, College of Animal Science and Technology, Sichuan Agricultural University, Chengdu, China; 3 Animal Disease Prevention and Green Development Key Laboratory of Sichuan Province, College of Life Sciences, Sichuan University, Chengdu, China; 4 National Key Laboratory for Swine Genetic Improvement and Germplasm innovation Technology, Jiangxi Agricultural University, Nanchang, China; Dartmouth College Geisel School of Medicine, UNITED STATES OF AMERICA

## Abstract

Copper is essential for reproductive function, yet its accumulation can lead to cytotoxicity and cuproptosis. However, the specific molecular mechanisms underlying granulosa cell cuproptosis and follicular atresia remain unclear. Particularly, the molecular pathway by which tRNA-derived fragments (tRFs), recognized as crucial epigenetic regulators, are involved in the regulation of granulosa cell cuproptosis requires further elucidation. In this study, we indicated that copper accumulation disrupted mitochondrial respiration and protein lipoylation, resulting in impaired mitochondrial TCA cycling and subsequent cellular metabolic imbalance. Furthermore, a direct correlation was identified between tRFs and copper homeostasis. Functional analysis demonstrated that tRF-Gly-M3, produced by angiopoietin (ANG) splicing, was significantly upregulated in granulosa cells cuproptosis, and impaired mitochondrial function to induce cuproptosis by silencing the expression of GLS mRNA. tRF-Gly-M3 in exosomes secreted by cuproptosis-induced granulosa cells was high expression, and these exosomes could be delivered into oocytes. tRF-Gly-M3 also impaired oocytes mitochondrial metabolic function, inhibited oocytes maturation, first polar body extrusion and parthenogenesis via silencing GLS mRNA. Overall, our findings indicated that tRFs from granulosa cells could be intercellularly delivered to oocytes, effectively regulating oocyte development.

## Author summary

Copper is vital for healthy reproduction, but excessive copper levels can poison cells, especially the critical granulosa cell in the ovary that support developing oocytes. Until now, the exact process of how this copper-induced stress causes cell death and impairs fertility has remained a mystery. We discovered that excess copper paralyzes the energy-producing centers of these granulosa cells, causing a massive energy failure. In response to this stress, we show that the cells increase production of tiny RNA fragments. These fragments directly shut down a crucial gene required for energy metabolism, forcing the granulosa cells cuproptosis. Crucially, we found that the granulosa cell exosomes package these RNA fragments and send them directly into oocytes. This external delivery then wreaks havoc on the oocytes’s own energy centers, severely blocking its ability to mature and develop properly. By uncovering this unique communication pathway where damage signals are transmitted between cells, our findings offer new insights into how environmental heavy metal pollution can harm fertility and suggest new directions for protecting reproductive health.

## 1 Introduction

The ovarian reserve, defined by the population of primordial follicles, is the primary determinant of a woman’s reproductive lifespan. This reserve is maintained through the tightly regulated processes of follicular development, activation, and ovulation [1]. Disruptions in follicular growth or uncontrolled activation may result in disorders such as atresia, premature ovarian failure, polycystic ovary syndrome, and infertility, influenced by genetic and environmental factors [2,3]. Follicular atresia, a degenerative process occurring at any developmental stage, is primarily induced by granulosa cell (GCs) apoptosis and significantly contributes to diminished female fertility [4]. Thus, programmed cell death is vital for ovarian development [5], with apoptosis occurring in both fetal and adult follicles. In fetuses, apoptosis primarily affects oocytes, while in adults, it predominantly targets granulosa cells in secondary and cystic follicles [6]. Oocyte development and maturation take place within an environment surrounded by granulosa cells, where adjacent granulosa cells differentiate into cumulus cells (CCs), engaging in bidirectional communication with the oocyte [7,8]. Understanding the interaction mechanisms between granulosa cells and oocytes is essential for regulating follicular development and oocyte maturation.

Metals are pervasive in daily environments, with the human body absorbing them via food, inhalation, and skin contact. Prolonged low-dose exposure or short-term high-dose exposure can result in metal accumulation within the body. Notably, various metals have been detected in follicular fluid [9]. Importantly, copper is an essential trace element that serves as a catalytic cofactor in various physiological processes, such as energy metabolism, mitochondrial respiration, and antioxidation. Although copper levels in cells are typically maintained at low concentrations, slight increases can induce oxidative stress and impair cellular function. Accumulation of copper results in the aggregation of mitochondrial ester acylated proteins and destabilizes iron-sulfur cluster proteins [10], resulting in proteotoxic stress and subsequent cell death, particularly affecting ovarian granulosa cells [11–13]. Our earlier study identified a potential link between cuproptosis and 3-NP-induced oxidative stress in granulosa cells [20]. It implied that copper could play a significant role in follicular development, with granulosa cell death being crucial in abnormal follicular development and ovarian dysfunction [14]. Nonetheless, the precise mechanism by which cuproptosis induces ovarian damage is yet to be elucidated.

Transfer RNA⁃derived small RNAs (tRFs) are a novel class of small noncoding RNAs, 18–34 nucleotides in length, generated through targeted cleavage of mature or precursor tRNAs (pre-tRNAs) [15]. Emerging evidence suggested that tRFs exhibited elevated expression and marked specificity under distinct physiological or pathological states, which might be modulated by various stress conditions, including oxidative stress, starvation, and heat shock [4,16]. tRFs interact with mRNAs or proteins to influence gene expression, translation, and epigenetic modifications [17]. These features position them as promising regulators of biological processes and potential biomarkers [18]. Notably, tRFs have emerged as critical regulators of ovarian function, mediating granulosa cell programmed death to modulate ovarian physiology. For instance, Huang et al. demonstrated that tsRNA-3043a promoted premature ovarian aging by targeting FLT1 to enhance apoptosis and senescence of ovarian granulosa cells [19]. Additionally, tRF-Gly-GCC induced follicular atresia by inhibiting granulosa cell proliferation and promoting granulosa cell ferroptosis through downregulation of MAPK1 [5]. However, the role of tRFs in the regulation of follicular development by copper homeostasis remains underexplored.

Previously, we identified a correlation between oxidative stress-induced apoptosis in granulosa cell and cuproptosis. In this study, we found that copper initiated the cuproptosis pathway by inducing mitochondrial oxidative stress, disrupting glutathione metabolic homeostasis, and leading to mitochondrial metabolic disorders. Additionally, we established a direct correlation between tRNA-derived fragments (tRFs) and the pathogenesis of cuproptosis.

## 2 Restults

### 2.1 Follicular atresia is associated with copper overload induced cuproptosis in pig

**Fig 1:**
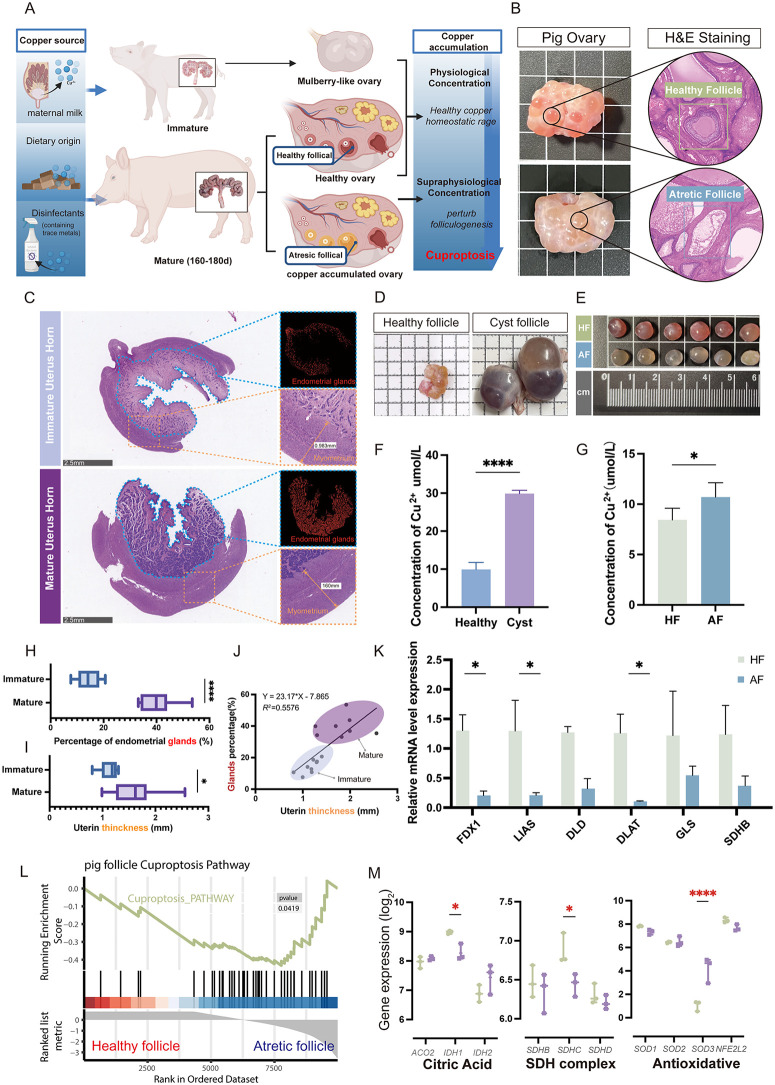
Follicular atresia is associated with copper overload induced cuproptosis in sows. **(A)** Schematic illustration of copper intake sources and the mechanism by which excessive copper accumulation in the ovary induces follicular atresia; Created in BioRender. Wu, **S.** (2026) https://BioRender.com/c7v99i5. **(B)** Morphology of ovarian tissue containing both healthy and atretic follicles, and corresponding hematoxylin and eosin (HE) staining; **(C)** HE staining of the sow uterine horn, with glandular areas marked in red using ImageJ. Scale bar = 200 μm; **(D)** Representative images comparing ovaries with healthy follicles (HF) and those with atretic follicles (AF); **(E)** Representative images of healthy follicles (HF) and atretic follicles (AF); **(F)** Measurement of copper ion concentration in follicular fluid from healthy and cystic follicles; **(G)** Comparison of copper ion concentration between HF and AF follicular fluid; **(H)** Quantitative analysis of gland numbers in the uterine horn; **(I)** Endometrial thickness (mm); **(J)** Pearson correlation analysis between myometrial thickness and the proportion of endometrial glands; **(K)** RT-qPCR analysis of mRNA expression levels of key cuproptosis regulatory genes in HF and AF; **(L)** Gene enrichment analysis from transcriptome sequencing of HF and AF; **(M)** RNA-seq identification of genes related to mitochondrial citrate metabolism, SDH subunits, and antioxidant defense. The data in **(E)** - (K) represents the means ± SD of ≥3 three times biological experiments. All *p* values were determined using a Student’s test; *p < 0.05, ****p < 0.0001.

Previous studies have shown that copper overload induced granulosa cell dysfunction and promote follicular atresia in the ovary [20,21]. We proposed the regulatory mechanisms in an animal model of copper exposure through diet (Fig 1A). Compared to healthy follicles (HF), atretic follicles (AF) exhibited zona pellucida wrinkling, granulosa cell degeneration, oocyte apoptosis, and nuclear consolidation (Fig 1B). Sexual maturation in sows is intricately linked to follicular development, we investigated uterine changes during this process. HE staining demonstrated a significantly greater myometrial thickness in sexually mature sows compared to their immature counterparts (Fig 1C and 1H). Additionally, the number of endometrial glands was notably higher in mature sows (Fig 1I). Pearson correlation analysis further indicated a relationship between myometrial thickness and the number of endometrial glands (R^2^ = 0.5576), suggesting a synergistic change between these two indices (Fig 1J). Subsequently, we collected ovaries from sexually mature sows, isolating follicles to distinguish between healthy, atretic, and cystic types based on morphology, diameter, internal structure, and vascular distribution. Cystic follicles were larger, with thin walls, grayish-white or grayish-red inner walls, and clear or bloody fluid, compared to healthy follicles. Atretic follicles exhibited smaller diameters, irregular shapes, turbid fluid, and reduced vascularity (Fig 1D-1E). Comparative pathological modeling further revealed characteristic copper ion enrichment in both atretic and cystic follicles (Fig 1F-1G). RT-qPCR analysis revealed significant downregulation of key cuproptosis regulators in atretic follicles (Fig 1K). Further GSEA analysis of RNA-seq data from healthy and atretic follicles indicated that differential gene expression in atretic follicles was enriched in cuproptosis-related pathways (Fig 1L). Furthermore, the expression of key regulators in the citric acid cycle, succinate dehydrogenase subunits, and antioxidant pathways were significantly altered in atretic follicles (Fig 1M). These findings indicated a potential regulatory role of copper ions in ovarian follicle development.

### 2.2 Copper overload directed towards the ovary in mouse model is linked to cuproptosis

**Fig 2:**
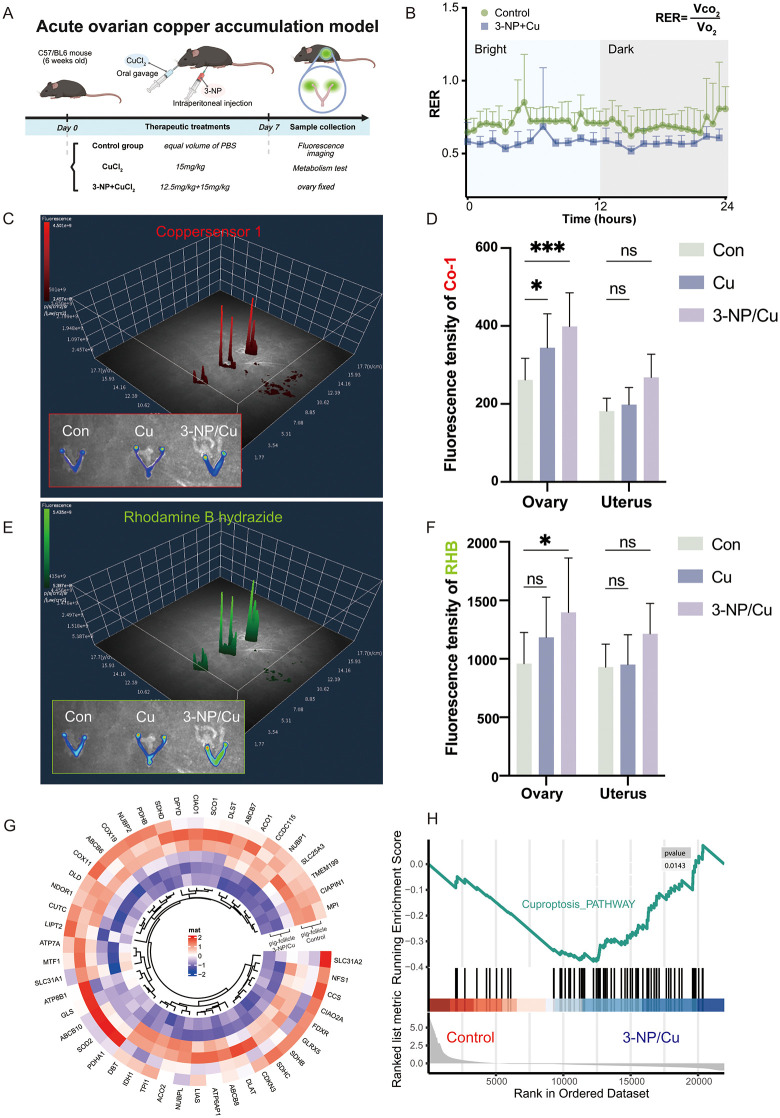
Ovarian targeting of copper overload in mice. **(A)** Schematic diagram of the construction of the mouse copper accumulation induction mode. Created in BioRender. Wu, **S.** (2026) https://BioRender.com/od6waou; **(B)** Metabolic cage analysis of the respiratory exchange rate (RER) in mice; **(C-D)** Three-dimensional visualization of monovalent copper content in mouse ovaries and uteri (C) and quantification of its fluorescence intensity **(D)**; **(E-F)** Three-dimensional visualization of divalent copper content in mouse ovaries and uteri (E) and quantification of its fluorescence intensity **(F)**; **(G)** Comparative transcriptomic analysis of follicles from control and copper-treated sows identified differentially expressed genes associated with copper-induced effects; **(H)** Gene enrichment analysis of transcriptomes from copper death-treated follicles. The data in (D) and (F) represents the means ± SD of ≥3 three times biological experiments. All *p* values were determined using a Student’s test; *p < 0.05, ***p < 0.001.

To validate the key target organs for copper toxicity, the study constructed a copper accumulation mouse model. Six-week-old mice were treated using the method outlined in Fig 2A. The 3-Nitropropionic acid (3-NP) is a powerful inducer of reactive oxygen species (ROS), and oxidative stress in the respiratory system can impair lung gas exchange and the respiratory exchange rate. In our study, treatment with 3-NP/Cu significantly reduced the respiratory exchange rate in mice (Figs 2B and S1B), indicating potential disruption of metabolic pathways. Subsequently, mice in the copper accumulation model were injected with a monovalent fluorescent copper ion probe (Coppersensor 1) and a divalent fluorescent copper ion probe (Rhodamine B hydrazide) via the tail vein. In vivo three-dimensional fluorescence imaging of the mice was performed to observe the changes in fluorescence intensity over time (S1A Fig). The results indicated that the fluorescence intensity in Cu-treated mice peaked after 2 hours (S1E Fig). Mice treated with both 3-NP and copper (3-NP/Cu) showed higher fluorescence intensity compared to mice treated with Cu alone (S1C-S1D Fig). The distribution of copper ions in key organs involved in basal copper metabolism, such as the liver, brain, and intestines, was examined using a 3D fluorescence presentation technique (S1F Fig). Significant monovalent copper-enriched signals were detected in ovarian tissues in the copper gavage (Cu) group and the oxidant injection plus copper gavage (3-NP/Cu) group (Fig 2C-2D). Furthermore, the 3-NP/Cu group exhibited significantly elevated divalent copper-enriched signals in the ovaries (Fig 2E-2F). These findings suggest that copper ions exhibit targeted enrichment in the ovary under conditions of copper overload and disruption of oxidative homeostasis.

To further explore copper overload’s impact on ovarian function, normal swine follicles were co-treated with 3-NP and copper, followed by RNA-seq analysis. The results revealed downregulation of 48 genes associated with the cuproptosis pathway in the 3-NP/Cu-treated group (Fig 2G). GSEA analysis further indicated that the differentially expressed genes in the 3-NP/Cu group were enriched in the cuproptosis pathway (Fig 2H), suggesting copper mutation may disrupt follicular gene expression, contributing to ovarian function abnormalities.

### 2.3 Copper hinders mitochondrial TCA cycling enzyme activity in granulosa cells

**Fig 3:**
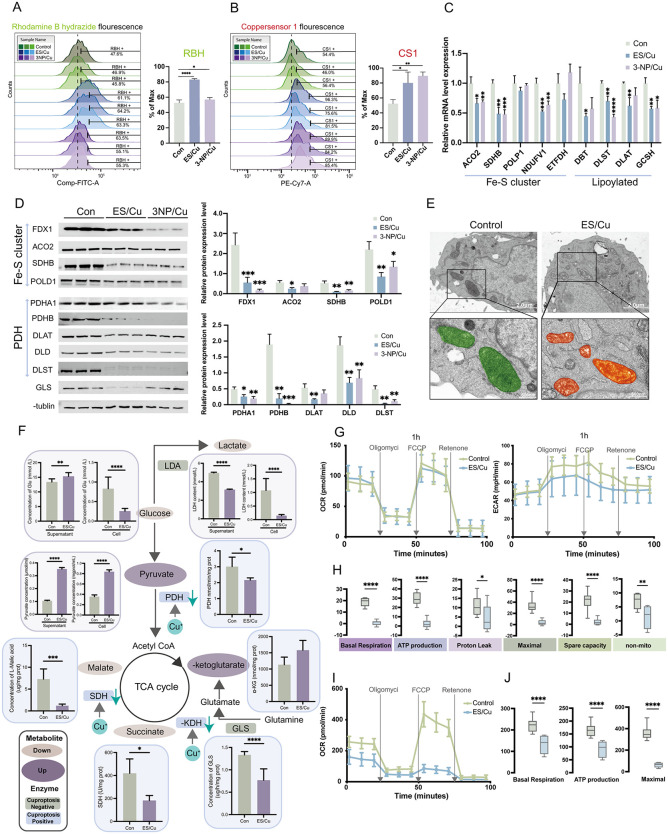
Copper impairs TCA cycle enzyme activity in granulosa cells. **(A)** Flow cytometry analysis of divalent copper ion accumulation in granulosa cells (left) and corresponding fluorescence intensity quantification (right); **(B)** Flow cytometry analysis of monovalent copper ion accumulation (left) and fluorescence intensity quantification (right); **(C)** RT-qPCR analysis of mRNA levels for iron-sulfur cluster proteins and lipid acylation modification proteins; **(D)** Western blot analysis of Fe-S cluster proteins and pyruvate dehydrogenase (PDH) subunit expression levels; **(E)** Mitochondrial transmission electron microscopy (Magnification, × 7.0k, 2μm, × 20.0k, 500nm); **(F)** Enzymatic activities of key TCA cycle enzymes (pyruvate dehydrogenase, α-ketoglutarate dehydrogenase, and succinate dehydrogenase) and levels of metabolites (lactate, glucose, pyruvate, malate) in granulocytes; **(G)** Hippocampal technique measures mitochondrial oxygen consumption rate (left) and extracellular acidification rate (right) after 1 hour of ES/Cu treatment; **(H)** Mitochondrial stress test: basal respiration, ATP production, electron leakage rate, maximal respiration, spare respiratory capacity, and non-mitochondrial respiration; **(I)** Hippocampal technique determines mitochondrial oxygen consumption rate after 12 hours of ES/Cu treatment; **(J)** Quantitative analysis of mitochondrial basal respiration, ATP production, electron leakage rate, maximal respiration, alternate respiratory capacity, and non-mitochondrial respiration in mitochondrial stress tests. The data in **(C)**–(F) represents the means ± SD of ≥3 three times biological experiments. All *p* values were determined using a Student’s test; *p < 0.05, **p < 0.01, ***p < 0.001, ****p < 0.0001.

Granulosa cells, as a key factor influencing follicular development, Granulosa cells, pivotal in follicular development, were exposed to the copper ion carrier ES and the oxidative stress inducer 3-NP, supplemented with copper ions, to investigate copper accumulation in these cells. Results indicated that both 3-NP/Cu and ES/Cu treatments markedly elevated Cu^+^ and Cu^2+^ levels in granulosa cells (Fig 3A-3B). ES, a lipid-soluble copper ion carrier, encapsulated Cu^2+^ and promoting its cellular uptake. RBH staining demonstrated that co-supplementation with the copper ion carrier ES and Cu facilitated Cu^2+^ translocation into cells (S2A-S2B Fig), which is consistent with the study of Han et al [22]. Additionally, coppersensor-1 staining revealed that the combined supplementation of ES and 3-NP enhanced intracellular Cu^+^ accumulation (S2C-S2D Fig). These observations indicate that a similar copper accumulation phenomenon occurs in granulosa cells. High copper concentrations destabilize iron-sulfur (Fe-S) proteins and cause aggregation of lipoylation proteins. Analysis of mRNA expression and protein levels revealed that both mRNA and protein levels of Fe-S genes were decreased in ES/Cu- and 3-NP/Cu-treated granulosa cells, with a similar reduction observed for lipoylation-related genes (Fig 3C and 3D). The combined dysregulation of lipid acylation and iron-sulfur cluster proteins led to severe metabolic disturbances and proteotoxic stress, culminating in copper-induced cell death. Mitochondria are recognized as the primary target of cuproptosis, and the research indicated that cuproptosis ensues upon mitochondrial impairmen [23]. Mitochondrial cristae exist in two primary forms: lamellar and tubular. Lamellar cristae are predominantly involved in oxidative phosphorylation to supply ATP, while tubular cristae are linked to hormone synthesis, metabolic activity, and signaling (S2E Fig). Both types were identified in granulosa cells (S2F Fig). Following copper overload treatment, ES/Cu group resulted in a swollen mitochondrial matrix, reduced and disorganized cristae, and vacuolization (Fig 3E), indicating mitochondrial damage.

Mitochondrial damage induces metabolic disorders. Excessive copper levels have been shown to induce the oligomerization of pyruvate dehydrogenase (PDH), α-ketoglutarate dehydrogenase (α-KDH), and succinate dehydrogenase (SDH)-associated fatty acylated protein subunits within the mitochondrial tricarboxylic acid (TCA) cycle, leading to mitochondrial metabolic dysregulation [24,25]. Hence, we investigated the activity of pivotal regulatory enzymes and the levels of essential metabolites within the TCA cycle pathway. Our findings revealed a significant decrease in the activities of PDH, α-KDH, and SDH enzymes following copper overload. Impeding the mitochondrial TCA cycle hinders glucose uptake and utilization. Inhibiting pyruvate utilization elevates pyruvate levels. Reduced succinate dehydrogenase (SDH) enzyme activity decreases downstream malic acid content (Fig 3F). This observation suggests that copper overload may lead to imbalance of TCA cycle and eventually induce cuproptosis. To assess the dependence of cuproptosis on mitochondrial function, we examined the impact of copper overload on the regulation of mitochondrial respiratory metabolism in granulosa cells. Following 1h and 12h of ES/Cu treatment, both oxygen consumption rate (OCR) and extracellular acidification rate (ECAR) exhibited significant reductions, indicative of mitochondrial stress. Parameters such as basal respiration, ATP production, electron leakage rate, maximal respiration, spare respiratory capacity, and non-mitochondrial respiration demonstrated characteristics of a low-energy bioenergetic state (Fig 3G-3J). Therefore, we postulated that copper overload in granulosa cells (GCs) might be linked to copper synucleinopathy, resulting in mitochondrial dysfunction and perturbation of the TCA cycle.

### 2.4 Cuproptosis induces distinct gene expression patterns and metabolic irregularities in follicular

**Fig 4:**
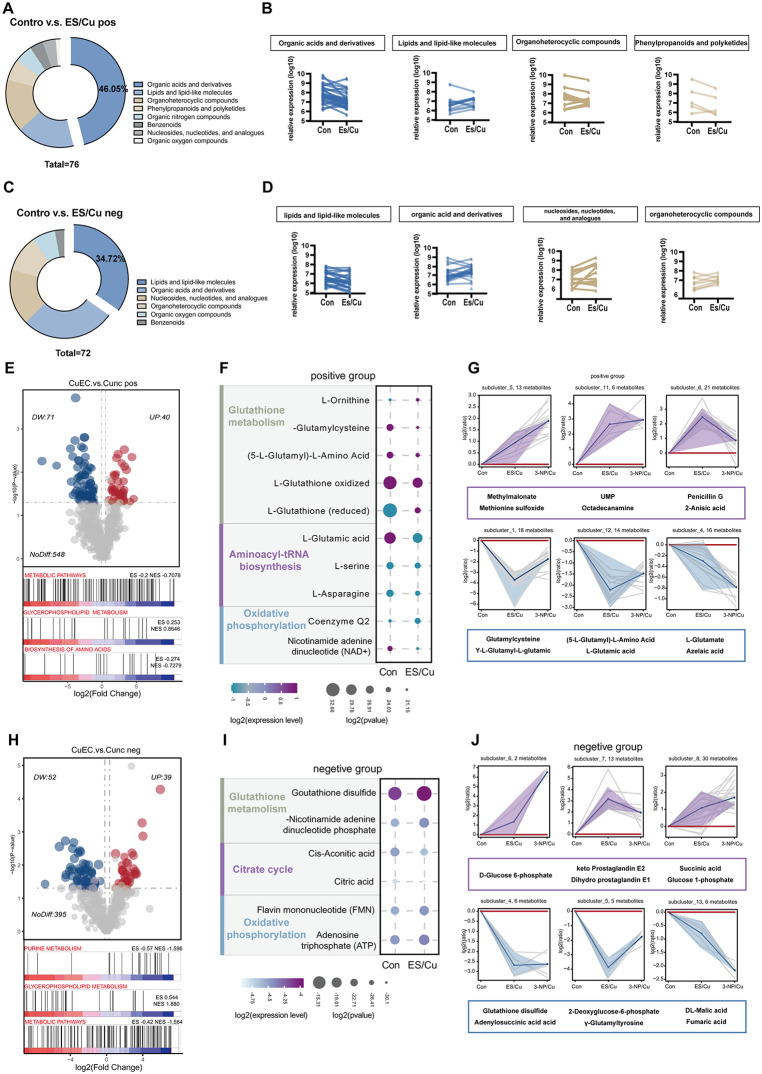
Differential metabolite analysis of follicular cuproptosis. **(A)** Pie chart of metabolite species in positive ion mode; **(B)** Differential metabolites in positive ion mode; VIP (Variable Importance in the Projection) > 1.0, FC > 1.2, or FC < 0.833, *P* < 0.05; **(C)** Pie chart of metabolite species in negative ion mode; **(D)** Differential metabolites in negative ion mode; VIP (Variable Importance in the Projection) > 1.0, FC > 1.2, or FC < 0.833, *P* < 0.05; **(E)** Metabolite volcano plots and GSEA enrichment plots of KEGG pathways in positive ion mode; **(F)** Bubble plots for GO enrichment analysis of metabolites in positive ion mode; **(G)** K-means analysis of granule cell metabolite subclasses in positive ion mode, showing three up-regulated (purple) and three down-regulated (blue) subclasses in Copper-treated groups (ES/Cu and 3-NP/Cu); **(H)** Metabolite volcano plots and GSEA enrichment plots of KEGG pathways in negative ion mode; **(I)** Bubble plots for GO enrichment analysis of metabolites in negative ion mode **(J)** K-means analysis of granulocyte metabolite subclasses in negative ion mode, with three up-regulated (purple) and three down-regulated (blue) subclasses in the copper death-treated group (ES/Cu and 3-NP/Cu).

Previous findings indicated that copper overload in granulosa cells, leading to cuproptosis, might result in significant metabolic disorders. LC-MS/MS analysis of ES/Cu-treated mouse follicles revealed alterations in metabolites associated with organic acid, lipid, and amino acid metabolism in both positive and negative ion modes (Fig 4A and 4C). Compared to the control group, the expression of these metabolites differed significantly in the ES/Cu-treated group (Fig 4B and 4D). Subsequent metabolomic profiling identified 111 and 91 differentially abundant metabolites in the positive and negative ion modes respectively. Gene set enrichment analysis (GSEA) revealed that the differential metabolites were enriched in metabolism-related pathways, including glycerophospholipid metabolism and purine metabolism (Fig 4E and 4H).

Further, Gene Ontology (GO) functional enrichment analysis revealed significant enrichment of the Glutathione metabolism, Aminoacyl-tRNA biosynthesis, and Oxidative phosphorylation pathways in the positive ion mode (*P* < 0.05). Additionally, key metabolites within these pathways, including glutamylcysteine, reduced glutathione (GSH), glutamate, and nicotinamide adenine dinucleotide (NAD+), exhibited marked decreases (Fig 4F). In negative ion mode, the TCA cycle exhibited significant enrichment. Notably, the levels of cis-aconitic acid and citric acid were markedly decreased, whereas oxidized glutathione (GSSG) and nicotinamide adenine dinucleotide phosphate (NADP+) levels increased significantly (Fig 4I). Using k-means cluster analysis, the follicular metabolic characteristics during copper-induced cell death were elucidated, revealing copper death-specific metabolic profiles. These profiles showed significant variations in the expression of key intermediates in the glutathione metabolic pathway, RNA biosynthesis, glycolysis, and the TCA cycle (Fig 4G and 4J). The above results suggest that cuproptosis disrupts follicular metabolic homeostasis, ultimately leading to ovarian dysfunction.

### 2.5 Cuproptosis is associated with aberrant tRFs expression in granulosa cells

**Fig 5:**
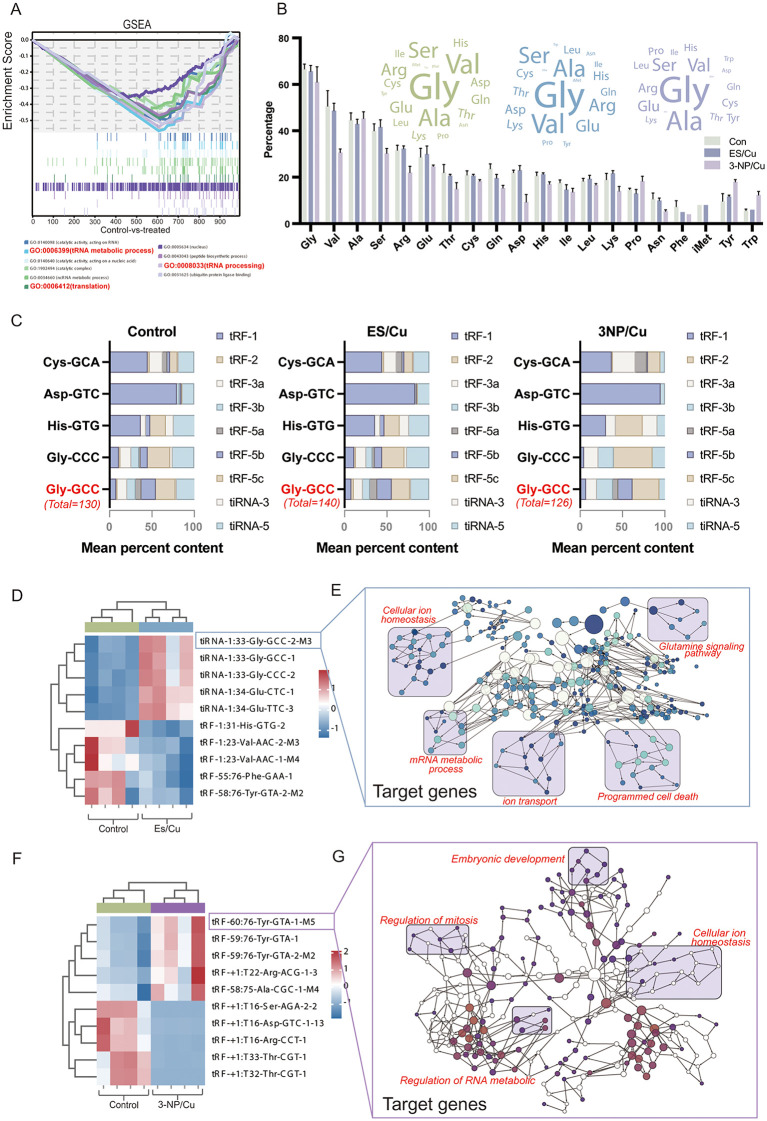
Differential tRFs expression in granulosa cells with copper accumulation. **(A)** GSEA enrichment analysis of RNA-seq data for differentially expressed genes in granulosa cells subjected to copper-induced stress; **(B)** Graph illustrating the number and percentage of tRF precursor sources in granulosa cells; **(C)** Distribution of differentially expressed tRFs isoforms; **(D)** Heat map depicting differentially expressed tRFs in granulosa cells under ES/Cu treatment; **(E)** KEGG analysis and pathway clustering for target genes of tiRNA-1:33-Gly-GCC-2-M3 (tRF-Gly-M3); **(F)** Volcano plot and heat map of differentially expressed tRFs in granulosa cells under 3-NP/Cu treatment; **(G)** KEGG analysis and pathway clustering for target genes of tRF-60:75-Tyr-GTA-1-M5.

To investigate the molecular mechanisms and pathways of mitochondrial damage triggered by cuproptosis, we conducted a GSEA gene enrichment analysis on differentially expressed genes. The analysis revealed significant enrichment of the tRNA metabolic process, tRNA function, and tRNA translation pathway in cuproptosis granulosa cells (Fig 5A). tRNA-derived fragments (tRFs), produced by cleavage at specific sites of tRNA or pre-tRNA, are vital in stress response, signal transduction, and gene expression. To further clarify the regulatory mechanisms of tRFs in cuproptosis, high-throughput sequencing was employed to analyze tRFs expression profiles in cuproptosis follicles. The tRNA categories of differential tRF sources (Fig 5B) and the distribution of differential tRF isoforms (Fig 5C) were quantified. Notably, the tRFs exhibiting the most pronounced expression changes predominantly originated from glycine (Gly)-associated tRNAs, with tRFs corresponding to the Gly-GCC codon showing a marked increase in expression.

Further, the present study identified Differentially expressed tsRNAs were identified in two copper-induced cell death models (fold change ≥ 1.5, *P* < 0.05), followed by heat map analysis of these tRFs (Fig 5D-5E, left). The most significantly differentially expressed tRF was selected for further KEGG enrichment analysis. The results showed that the enrichment of differentially expressed tRF target gene pathways in both cuproptosis models was associated with cellular metal metabolism and RNA metabolism (Fig 5D-5E, right). Notably, the target gene of tiRNA-1:33-Gly-GCC-2-M3 (tRF-Gly-M3), which was highly expressed in the ES/Cu-treated group, was enriched in the glutamine metabolism signaling pathway, aligning with our earlier metabolome sequencing results (Fig 4). Within mitochondria, glutaminase converts glutamine into glutamate and ammonia. The generated glutamate is further metabolized into α-ketoglutarate (α-KG) through the action of glutamate dehydrogenase (GLUD1) or transaminases (GOT, GPT), ultimately entering the tricarboxylic acid cycle (TCA) for energy production. This implies that tRF may regulate copper metabolism by affecting mitochondrial function through the glutamine metabolic pathway.

### 2.6 tRF-Gly-M3 is the critical epigenetic factor regulating granulosa cells cuproptosis

**Fig 6:**
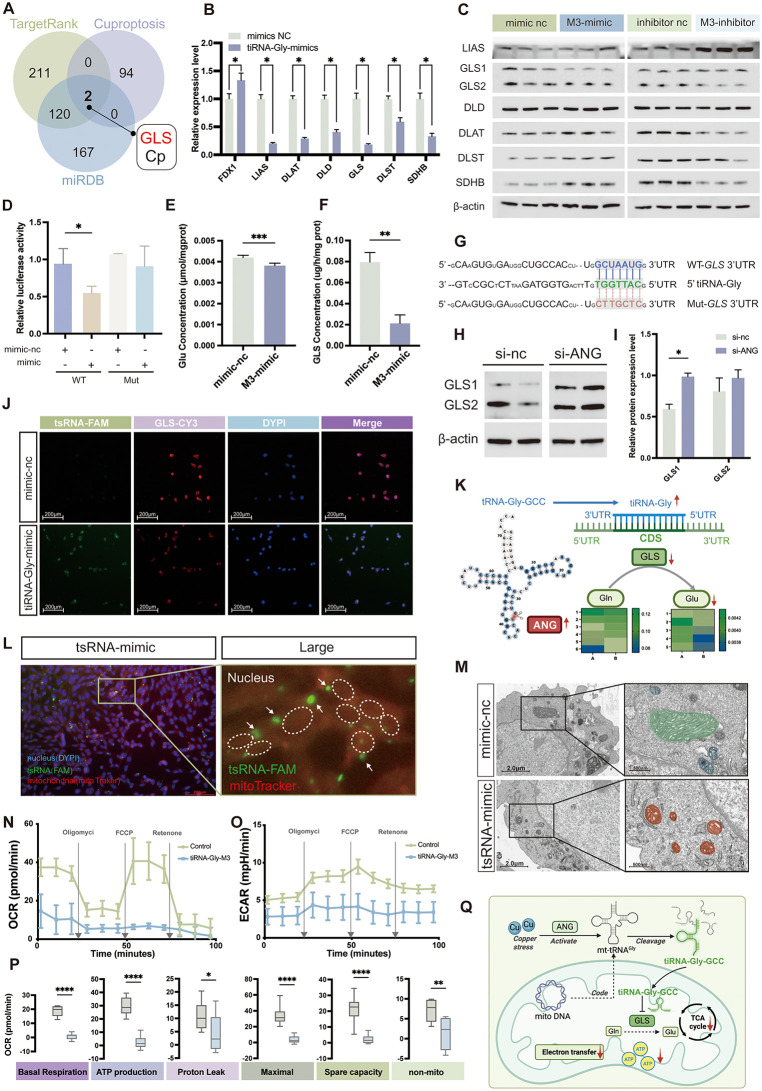
tRF-GLS regulates the cuproptosis pathway. (A) Venn diagram analyses target genes of tRF-Gly-M3; (B) RT-qPCR analyses the mRNA expression levels of key cuproptosis genes in granulosa cells; (C) Western blot analyses the protein expression of target genes and key cuproptosis genes after transfer tRF-Gly-M3 mimics and inhibitors; (D) Dual-luciferase assay analyses the targeting relationship between tRF-Gly-M3 and GLS; (E-F) Measurement of mitochondrial glutamate content (E) and GLS enzyme activity in granulosa cells post-transfection with tRF-Gly-M3; (G) Prediction of tRF-Gly-M3 binding sites on target genes and mutation sites; (H-I) Western blot analyses the GLS protein expression (H) and quantification of protein levels (I) following ANG knockdown; (J) FISH assay analyses the RNA level in tRF-Gly-M3 (green, FAM probe) and GLS (red, CY3 probe) post-transfection; Scale bar  =  200 μm; (K) Diagram illustrating the precursor tRNA splicing site of tRF-Gly-M3 and its gene-targeting regulatory mechanism; (L) FISH assay examines the cellular localization of tRF-Gly-M3, mitochondria marked in red (Mitotracker), tRF-Gly-M3 in green (FAM probe), the nucleus highlight in the white box; Magnification,  ×  100); (M) Transmission electron microscopy of mitochondria 24 hours post-transfection with mimic-nc and tRF-mimic; (O) Hippocampal technique measures extracellular acidification rate; (P) Mitochondrial stress test assesses mitochondrial basal respiration, ATP production, electron leak rate, maximal respiration, spare respiratory capacity, and non-mitochondrial respiration; (Q) Schematic diagram illustrating mitochondria-derived tRFs in mitochondrial regulatory mechanisms. Created in BioRender. Wu, S. (2026) https://BioRender.com/xzpyplq. The data in (B)–(F) represents the means  ±  SD of ≥3 three times biological experiments. All p values were determined using a Student’s test; *p  <  0.05, **p  <  0.01, ***p  <  0.001, ****p  <  0.0001.

Based on the above findings, we investigated the role of tRF-Gly-M3 in cellular cuproptosis. Initially, we identified tRFs with elevated expression under ES/Cu treatment, aligning with RNA-seq data (S3A Fig). tRFs function analogously to microRNAs, binding to Argonaute (Ago) proteins and targeting complementary mRNA sequences to mediate post-transcriptional regulation [26–28]. To elucidate the mechanism by which tRF-Gly-M3 exerts its biological effects in granulosa cells, we integrated RNA-seq transcriptome data with TargetRank and miRDB analyses. This screening approach identified glutaminase (GLS) and ceruloplasmin (Cp) as target genes of tRF-Gly-M3 (Fig 6A). GLS comprises two isoforms, GLS1 and GLS2. Research indicates that GLS plays a role in mitochondrial glutamine metabolism by converting glutamine to glutamate, thereby supporting the TCA cycle and oxidative phosphorylation [29]. Furthermore, glutamate generated through GLS1 activity contributes to glutathione (GSH) synthesis, crucial for maintaining redox balance [30]. The glutathione metabolic pathway affected by cuproptosis, as discovered in the previous research, is closely mirrored by the findings. Consequently, we hypothesized that tRF-Gly-M3 exerts its biological functions by targeting glutaminase. Initially, we observed that the high expression of tRF-Gly-M3 in granular cells significantly impacted the cuproptosis regulatory pathway, notably suppressing the mRNA (Fig 6B) and protein levels of the target gene GLS (Figs 6C and S3B). Conversely, inhibition of tRF-Gly-M3 produced the opposite effect (Figs 6C and S3C). In addition, we observed that mitochondrial glutamine (Glu) levels decreased (Fig 6E) and glutaminase (GLS) activity was markedly inhibited (Fig 6F) following tRF-Gly-M3 overexpression, indicating that tRF-Gly-M3 may play a regulatory role by modulating mitochondrial GLS expression. Subsequently, we predicted the binding site of tRF-Gly-M3 to GLS and designed mutant sequences for the binding site (Fig 6G). Dual-luciferase reporter assays demonstrated that mutation of this binding site restored luciferase expression, compared to the wild-type GLS (Fig 6D). Furthermore, fluorescence in situ hybridization (FISH) confirmed the co-localization of tRF-Gly-M3 and GLS, further corroborating the target-binding relationship (Fig 6J). Since tRFs modulate gene expression by interacting with Ago family proteins, we specifically knocked down Ago1, Ago2, and Ago3 in granulosa cells. Only the co-treatment with Ago3 knockdown and tRF-Gly-M3 mimics restored GLS mRNA expression (S3D-S3I Fig). In addition, protein–RNA docking analysis (https://alphafoldserver.com/) indicated a binding interaction between tRF-Gly-M3 and AGO3 (S3M Fig). RIP–qPCR further confirmed that tRF-Gly-M3 binds specifically to endogenous AGO3 (S3N and S3O Fig). Together, these results suggest that tRF-Gly-M3 binds to Ago3, targeting GLS to suppress its expression.

tRNA splicing is primarily mediated by angiogenin (ANG), endonuclease Z (RNaseZ), and cytoplasmic homologous ribonuclease Z2 (ELAC2) [31,32]. Previous studies have indicated that tRF-Gly-M3 is cleaved by the endonuclease ANG at position 33 of tRNA [29,30], we found that knocking down ANG reduced the expression of tRF-Gly-M3 (S3J and S3L Fig) and impaired cell proliferation (S3K Fig). Notably, the protein expression level of GLS was significantly restored after ANG knockdown (Fig 6H). These findings suggested that ANG cleavage upregulated tRF-Gly-M3, which might then associate with the Ago3 protein to target and silence GLS, thereby influencing the regulation of the cuproptosis pathway (Fig 6K). To investigate the role of tRNA-Gly-M3, a product of ANG-mediated tRNA cleavage under cuproptosis, in regulating mitochondrial function through GLS. Firstly, fluorescence in situ hybridization (FISH) combined with mitotracker red staining revealed mitochondrial localization of tRF-Gly-M3 (Fig 6L). Significantly, cells overexpressing tRF-Gly-M3 exhibited disrupted and fewer mitochondrial cristae (Fig 6M), corroborating our earlier findings. Impaired mitochondrial function frequently manifests as disrupted respiratory metabolism [33]. To assess the metabolic state of cells with high tRF expression, we measured the mitochondrial oxygen consumption rate (OCR) and extracellular acidification rate (ECAR) using a cellular metabolic analyzer. The treatment group exhibited hallmarks of mitochondrial stress, with both OCR and ECAR significantly decreased (Fig 6N-6O). Furthermore, analysis of additional metabolic parameters, including basal respiration, ATP production, electron leakage, maximal respiration, spare respiratory capacity, and non-mitochondrial respiration, revealed an overall low-energy bioenergetic phenotype (Fig 6P). These revealed that tRF-Gly-M3 exerts a regulatory role analogous to that of ES/Cu in the modulation of mitochondrial respiratory metabolism. tRF-Gly-M3 may influence mitochondrial function by silencing GLS expression, thereby contributing to the regulation of cellular cuproptosis (Fig 6Q).

### 2.7 Granulosa cell mediates oocyte development through intercellular communication by exosome

**Fig 7:**
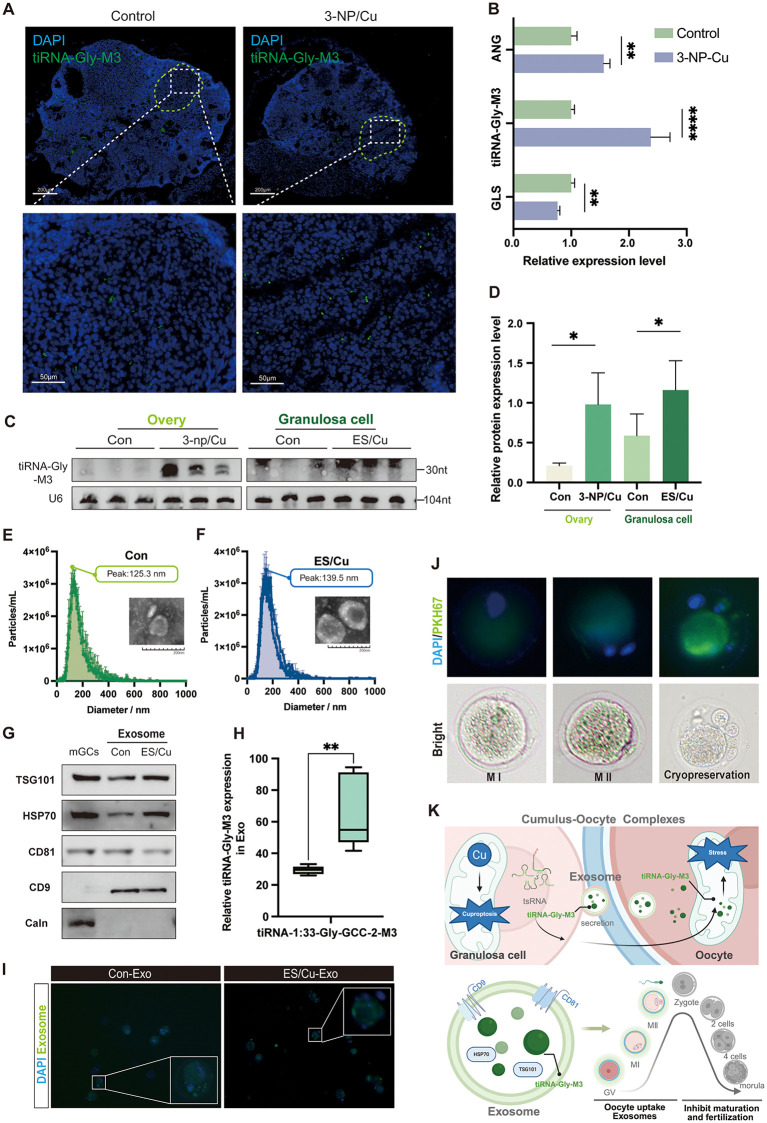
Granulosa cell-derived tsRNA is secreted and transferred to oocytes through the exosomal pathway. **(A)** FISH assay assesses tRF-Gly-M3 expression in mouse ovary tissues post-copper treatment (× 100, 200 μm; × 400, 50 μm;); **(B)** RT-qPCR analyses ANG, GLS mRNA, and tRF-Gly-M3 levels in mouse ovaries; **(C-D)** Northern blot analysis of tRFs levels in mouse ovaries (C) and granulosa cells (D) with quantification; **(E-F)** NAT and negative staining of exosomes from copper treatment; **(G)** Western blot analyses the marker protein expression of exosome; **(H)** RT-qPCR measures tRF-Gly-M3 levels in exosomes; **(I)** PKH67 staining of exosomes from granulosa cells co-cultured with oocytes; **(J)** PKH67 staining of exosomes with oocytes at different maturation stages; **(K)** Flowchart illustrating granulosa cell-derived tRF modulation of oocytes via exosomes. Created in BioRender. Wu, **S.** (2026) https://BioRender.com/1u3ozh2. The data in **(B)**–(D) represents the means ± SD of ≥3 three times biological experiments. All *p* values were determined using a Student’s test; *p < 0.05, **p < 0.01, ****p < 0.0001.

Under pathological conditions, high levels of tRF-Gly-M3 were detected in mouse ovarian follicles subjected to cuproptosis (Fig 7A). Quantitative analysis showed elevated tRF-Gly-M3 and ANG expression in the 3-NP/Cu group, with a concurrent decrease in GLS expression (Fig 7B), consistent with prior in vitro findings. Northern blotting corroborated these observations (Fig 7C-7D). Granulosa cells, the predominant cell type within the follicle, offer nutritional support to the oocyte and communicate with it via secretory factors and gap junctions to regulate oocyte maturation [7]. Material exchange between the oocyte and perifollicular follicles, including granulosa cells, occurs through extracellular vesicles during oocyte development, thereby modulating gene expression within the oocyte [34,35]. To elucidate the molecular mechanisms by which granulosa cells affect oocyte development, we isolated extracellular vesicles secreted by these cells. Using nanoparticle tracking analysis (NTA), we quantified the particles and confirmed their size distribution, consistent with the characteristics of exosomes. Additionally, electron microscopy negative staining validated the morphology and integrity of exosomes (Fig 7E-7F). Western blot analysis of exosome marker proteins further supported the presence of exosome components (Fig 7G). Notably, RT-qPCR assays demonstrated a significant upregulation of tRF-Gly-M3 in extracellular vesicles following ES/Cu treatment (Fig 7H).

Subsequently, granulosa cell exosomes from various treatments were co-cultured with oocytes, and successful entry of exosomes into oocytes was observed through PKH67 staining (Fig 7I). Furthermore, the co-cultivation of exosomes with oocytes at different maturation stages and cryopreserved oocytes also demonstrated exosome internalization (Fig 7J). Based on the above findings, we hypothesize that tRF-Gly-M3, expressed in granulosa cells, may be involved in the secretion of extracellular vesicles into oocytes, potentially triggering cuproptosis by causing mitochondrial dysfunction in oocytes (Fig 7K).

### 2.8 tRF-Gly-M3 plays a key role in regulating oocyte development

**Fig 8:**
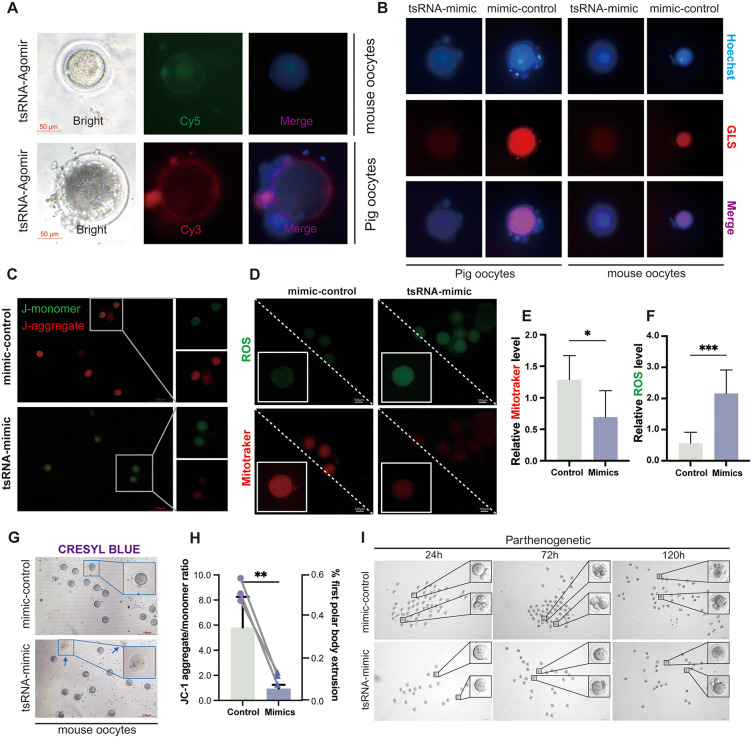
tsRNA plays a crucial role in regulating oocyte development by modulating mitochondrial activity. (A) Mouse and sow oocytes transfected with tRF-Gly-M3 agomir were visualized using mouse agomir-Cy5 (green), porcine agomir-Cy3 (red), and nuclei (blue); Scale bar  =  50 μm; (B) Immunofluorescence staining analyses GLS expression in swine and mouse oocytes transfected with tRF control and mimic after 24 hours; (C) Mitochondrial membrane potential in oocytes was assessed 24 hours after transfection with tsRNA control and mimic, JC-1 dimer (red), JC-1 monomer (green); Scale bar  =  100 μm (D-F) Reactive oxygen species (ROS) in oocytes were detected using ROS staining (green) and Mitotracker (red), with fluorescence intensities quantified for Mitotracker (E) and ROS (F); (G) leucocyanidin blue stains mouse oocytes after 24 hours transfection with mimic-nc and tsRNA-mimic; Scale bar  =  100 μm; (H) The aggregation/monomer ratio (C) and first polar body extrusion rate (G) were analyzed; (I) Representative images of blastocysts of parthenogenetic activation post-transfection with tsRNA control and mimic. The data in (E)–(K) represents the means  ±  SD of ≥3 three times biological experiments. All p values were determined using a Student’s test; *p  <  0.05, **p  <  0.01, ***p  <  0.001.

Oocyte maturation and development critically rely on mitochondrial energy, with studies highlighting mitochondrial inheritance as a pivotal factor in oocyte quality and zygote development [36,37]. To further explore tRF’s regulatory role in oocyte maturation, porcine and mouse follicles were obtained and subjected to in vitro maturation culture with tRF-agomir transfection. We observed that tRF-Gly-M3 successfully entered and was expressed in oocytes (Fig 8A). Immunofluorescence staining indicated that tRF-Gly-M3 overexpression inhibited GLS expression in oocytes (Fig 8B), suggesting its potential involvement in mitochondrial regulation via GLS. Subsequent membrane potential staining revealed that tRF-Gly-M3 overexpression reduced mitochondrial membrane potential in oocytes (Fig 8C and 8H) and increased ROS production (Fig 8D-8F).

In addition, we found that overexpression of tRF-Gly-M3 inhibited the exclusion of the first polar body in oocytes, blocking their development (Fig 8G and 8H). We further induced oocyte maturation from the metaphase I (MI) to metaphase II (MII) stage in vitro, simulating parthenogenetic activation. Remarkably, tRF-Gly-M3-agomir transfection significantly inhibited oocyte development at 24 hours, with this inhibitory effect on polar body extrusion persisting up to 120 hours (Fig 8I). In conclusion, these findings indicate that tRF-Gly-M3 may regulate oocyte development by modulating mitochondrial function.

## 3 Discussion

Oocyte development and maturation is a tightly regulated process, governed by systemic signals and factors within the oocyte’s microenvironment. The interaction between the oocyte and its surrounding granulosa cells is a key determinant of oocyte development and maturation. The oocyte stimulates granulosa cell proliferation and differentiation, while granulosa cells significantly influence oocyte development and maturation [7]. Granulosa cell proliferation affects follicular development, whereas their apoptosis can lead to follicular atresia. Programmed cell death (PCD) of granulosa cells is closely linked to follicular atresia. In particular, granulosa cell apoptosis contributes to ovarian ageing by promoting atresia; once apoptotic cells reach 10% of the population, follicles enter an atretic state, which impedes follicular development and oocyte maturation [38,39]. Other PCD modalities, including granulosa cell pyroptosis [40] and ferroptosis, may also compromise ovarian reserve and affect ovarian function via distinct pathways [41]. More recently, cuproptosis—a novel form of PCD triggered by excess copper—has been shown to induce granulosa cell death and thereby impair ovarian function [42]. In this study, we elucidated the molecular mechanism through which cuproptosis-induced differentially expressed tRF triggers copper-mediated cell death in granulosa cells by impeding GLS-induced mitochondrial dysfunction.

Our prior research demonstrated that the impairment of the antioxidant system and intracellular copper accumulation in granulosa cells induced by 3-Nitropropionic acid (3-NP) results in cuproptosis, ultimately impacting follicular development [20]. In this current study, we comprehensively investigated the mechanism underlying granulosa cell cuproptosis. Firstly, we confirmed that atretic follicles exhibit elevated copper ion levels, with differentially expressed genes enriched in pathways linked to cuproptosis. Subsequently, using a mouse model to simulate environmental copper overexposure, we demonstrated that short-term administration (7 days) of low-dose copper (15 mg/kg) leads to significant copper accumulation in organs. Yang et al. [13] demonstrated that female rats injected with Cu NPs for 14 consecutive days exhibited significant accumulation of Cu NPs in the ovaries, resulting in ovarian injury, disrupted sex hormone balance, and ovarian cell apoptosis. Our findings align with theirs, showing that elevated environmental copper levels led to notable accumulation of copper ions in the ovaries and uterus, as well as increased copper ion levels in the intestines, liver, and other vital organs associated with copper metabolism. Furthermore, metabolic cage assays and respiratory exchange rate (RER) analyses in mice revealed notable differences in the area under the curve, indicating that copper overload disrupts respiratory metabolism and oxidative homeostasis.

Cuproptosis occurs via direct interaction with lipoylated components of the TCA cycle, causing protein aggregation and loss of iron-sulfur cluster proteins, leading to proteotoxic stress and cell death [12,43]. Our findings align with this mechanism. In our study, the expression of lipoic acid-dependent Fe-S cluster assembly proteins, such as succinate dehydrogenase subunit SDHB, was significantly reduced following ES/Cu and 3-NP/Cu treatments. This was accompanied by a decrease in lipoylation of the PDH, α-KDH, and SDH complexes, impairing their enzymatic activities and disrupting the TCA cycle. Blockage of the TCA cycle results in metabolite accumulation within the mitochondrial matrix, leading to mitochondrial swelling and blurred cristae structures. Cuproptosis notably reduces the expression of key TCA cycle metabolites, including cis-aconitic acid, citric acid, DL-malic acid, and fumaric acid. These revealed a dual mechanism by which cuproptosis impairs cellular energy metabolism: it impairs the electron transport chain by inhibiting Fe-S cluster biosynthesis and directly compromises the structural integrity of the TCA cycle enzyme complex by disrupting lipid acylation modification.

Copper overload could disrupt various metabolic functions by binding to cellular molecules. It’s widely recognized that tRFs result from the specific splicing of tRNAs triggered by certain stresses [43]. The studies report that, under stress, tiRNAs produced by ANG (angiogenin) cleavage bind to cytochrome C (Cyt C) or other apoptosis-related proteins and thereby inhibit apoptotic signalling [44]. Moreover, multiple investigations have confirmed that tRFs have key roles in mRNA silencing, the regulation of protein synthesis, inhibition of apoptosis, intercellular communication and epigenetic regulation [45]. Our GSEA analysis of differentially expressed genes in cuproptosis revealed significant enrichment in tRNA metabolic processes, tRNA functions, and translational pathways. Molecular transcription of Seryl-tRNA synthetase and tRNA-Arg was known to respond to copper ion-induced stress [46,47]. In this study, tRFs produced in follicles during cuproptosis showed an increase in tRFs from tRNAs encoding tyrosine (Tyr), lysine (Lys), and proline (Pro), and a decrease in tRFs from tRNAs encoding valine (Val), threonine (Tha), and cystine (Cys). This study provides the first direct evidence that different types of tRFs may have distinct roles in cuproptosis, suggesting these findings are not coincidental. In addition, previous studies have shown that copper ions could alter the intracellular localisation of angiogenin (ANG), bind to or activate ANG directly, and regulate ANG transcription [48]. We identified a 3’tiRNA, tRF-Gly-M3, highly expressed in cuproptosis, arising from ANG-mediated cleavage. This fragment’s expression positively correlates with the cuproptosis phenotype. Our study thoroughly examined tRF-Gly-M3’s biological function and regulatory role in cuproptosis. Similar to microRNAs, tRFs can silence endogenous genes by binding to the 3’ UTR region of target mRNAs [26]. We confirmed that GLS is the target gene interacting with tRF-Gly-M3. The α-ketoglutarate (α-KG) produced by the GLS-catalyzed glutaminase pathway enters the mitochondrial TCA cycle, contributing to aerobic respiration anabolism and playing a crucial role in mitochondrial function regulation [47,49]. tRF-Gly-M3 disrupts glutamine metabolism by targeting and inhibiting GLS mRNA stabilization, thereby perturbing the TCA cycle and activating the cuproptosis signaling pathway. Additionally, it indirectly inhibits GSH synthesis, reducing cellular antioxidant capacity and the ability to bind free copper. This study establishes a regulatory network linking glutamine metabolic homeostasis, mitochondrial functional integrity, and programmed cell death in granulosa cells. These findings underscore the central role of mitochondria in regulating granulosa cell function.

Mitochondrial function in granulosa cells and their interactions with oocytes are crucial for normal oocyte development. However, it remains unconfirmed whether tRF from granulosa cells is released extracellularly to influence oocyte development. This study firstly investigated tRF’s potential role in intercellular regulation of oocyte development. During in vitro oocyte maturation, transfection of tRF-Gly-M3 significantly impaired oocyte maturation, first polar body extrusion, and mitochondrial activity, potentially through modulating GLS expression. Interestingly, cuproptosis-induced granulosa cells secreted exosomes with elevated levels of tRF-Gly-M3. These granulosa cell-derived exosomes were able to enter the oocyte, suggesting a mode of information exchange between the two cell types. This finding further indicates that tRF-Gly-M3 may regulate oocyte and granulosa cell function through intercellular communication and signal transduction mediated by exosomes. Consequently, exosomes should become a central focus of future research to clarify the regulatory mechanisms of tRFs.

## 4 Conclusions

This study demonstrates that granulosa cells undergo cuproptosis during follicular atresia, leading to disorganized mitochondrial metabolism and impairment of the tricarboxylic acid cycle, indicating that mitochondria are the switch for cuproptosis. Mechanistically, cuproptosis-induced differentially expressed tRF alters granulosa cell mitochondrial metabolism through the cuproptosis key factor GLS, inducing granulosa cell cuproptosis and inhibiting oocyte maturation in vitro. Understanding the molecular events behind follicular atresia is essential for elucidating the physiological processes involved in ovarian follicular development and dynamics. This study provides evidence for the trafficking of granulosa cell-derived exosomes into oocytes, yet the direct regulatory role of granulosa cell-derived tRF in oocyte development remains underexplored. Future research should aim to clarify the molecular mechanisms by which tRF affects oocyte development and explore its potential as a marker for regulating follicular development.

## 5 Materials and methods

### 5.1 Ethical approval

All experimental animals were ethically approved by the Animal Protection and Ethics Committee of Sichuan Agricultural University (Chengdu, China, Approval No. 20250032), and all procedures adhered to the welfare and ethical standards set by the university’s Animal Management Committee.

### 5.2 Animals’ treatments

Twenty-four 6-week-old female C57BL/6 mice were randomly divided into three groups (n = 8): (I) control (Con), (II) copper chloride (CuCl_2_) PBS solution gavage (Cu), and (III) 3-NP injected copper solution gavage (3-NP/Cu). Treatment followed established protocols [20]. After 7 days of continuous treatment, mice were euthanized via cervical dislocation. Ovarian tissues were collected, with half stored at -80°Cand the remainder fixed in 4% paraformaldehyde.

In vivo imaging in mice: Following mouse treatment, Coppersensor-1 (CS1, HY-141411, MedChemexpress, USA) and Rhodamine B hydrazide (HY-Y0016, MedChemexpress, USA) via tail vein injection, following the manufacturer’s guidelines. Cu^+^ and Cu^2+^ were then detected in mouse organs using a small animal live imaging system.

### 5.3 Follicle and oocyte collection and in vitro culture

Mouse oocyte isolation: Isolated the mouse ovaries and rinsed them with pre-warmed PBS. Using a stereomicroscope, punctured the ovarian surface follicles with a 1 mL syringe needle to release the cumulus-oocyte complexes (COCs).

Sow oocytes isolation: Mature sow ovaries were rinsed with pre-warmed phosphate-buffered saline (PBS), stored in insulated containers, and transported to the laboratory. Follicles measuring 3–6 mm in diameter were selected according to the method mentioned before [20]. Follicular fluid was aspirated, and cumulus-oocyte complexes (COCs) with multiple layers of unexpanded cumulus cells and uniformly dark cytoplasm were isolated. These COCs were digested for 3–5 minutes at 37°C in M2 medium with 0.1% hyaluronidase, and cumulus cells were gently removed. Following three washes, GV stage oocytes were selected.

Oocyte culture: Oocytes were washed in TCM-199 with 2 mg/mL BSA and placed in wells containing 500 μL of maturation medium (TCM-199 with 3.05 mM glucose, 0.91 mM sodium pyruvate, 0.57 mM cysteine, 0.1% polyvinyl alcohol, 10 ng/mL FSH, and 0.5 µg/mL LH) under paraffin oil. They were incubated at 38.5°C with 5% CO₂ for 42–44 hours, with the medium refreshed every 24 hours to monitor maturation. Subsequently, oocyte morphology and maturity were evaluated using stereomicroscope, and the percentage of first polar body expulsion was recorded.

### 5.4 Cell culture, transfection, and treatment

Mouse granulosa cells (mGCs) line was purchased from PriCells. Cells were cultured in a humidified 37°C, 5% CO_2_ incubator in DMEM medium (12491015, Gibco) supplemented with 10% (v/v) fetal bovine serum and 1% (v/v) penicillin/streptomycin. After overnight inoculation in 12-well plates, cells were treated with medium containing Elesclomol (STA-4783), CuCl_2_-2H_2_O (10125-13-0, Merck), and 3-NP (163031, Sigma-Aldrich) as per the experimental protocol. Hieff Trans Universal Transfection Reagent (Shanghai Yeasen Biotechnology Co., Ltd.) was used to transfect mGCs cells with constructs for small RNAs, tRF mimics, mimic-nc, and agomir, as detailed in S1 Table.

### 5.5 Cell counting kit-8 (CCK-8)

Cell proliferation was assessed via the CCK-8 method. Cells were seeded in 96-well plates, treated or transfected as per protocol, and incubated for 24 hours. Subsequently, 10 μL of CCK-8 reagent was added to each well and incubated for 1 hours. Absorbance at 450 nm was measured, and cell proliferation inhibition was calculated.

### 5.6 tRF sequencing

Total RNA was extracted from copper-treated and control cell samples, and small RNA libraries were constructed using the Illumina Small RNA Library Prep Kit. The libraries were sequenced on the Illumina NovaSeq 6000 platform and the raw data were quality-controlled using FastQC. Bowtie was employed for sequence alignment, and miRDeep2 was utilized for identification and quantification of tRFs. Differentially expressed tRFs were identified using a threshold of log2 fold change > 1 and adjusted *p*-value < 0.05. Functional annotation of the differential tRFs was performed through GO and KEGG pathway analysis.

### 5.7 RNA sequencing

Cuproptosis-treated and control cell samples were collected, and total RNA was extracted. Libraries were constructed using the TruSeq RNA Library Prep Kit (Illumina) and sequenced on the Illumina NovaSeq 6000 platform. The raw data were quality-controlled using FastQC, aligned with HISAT2, and analyzed for differential expression using DESeq2. Genes exhibiting differential expression between groups were identified based on *P* < 0.05 and FC > 2.0 criteria.

### 5.8 Untargeted metabolomics (LC-MS/MS)

Elesclomol/Cu-treated and control cell samples underwent untargeted metabolomics sequencing. LC-MS/MS analysis was conducted by Shanghai Biospectrum Co. The raw MS data were imported into XCMS for metabolite identification, normalized to total peak intensity, and analyzed using SIMCA-P for PCA and OPLS-DA. Metabolites with VIP values greater than 1 were further assessed using Student’s t-test at the univariate level. Metabolomics data were examined for differential metabolites by evaluating the multiplicity of differences and the differential contribution of OPLSDA model variables in multivariate statistical analyses. Additionally, hierarchical clustering of differential metabolites was conducted using the MetaboAnalyst database, and differential metabolites were visualized.

### 5.9 Content of copper

Following cell treatment, a copper ion probe fluorescence staining solution was applied. After 30 minutes, changes in intracellular copper content were assessed via flow cytometry or observed using a fluorescence microscope. Tissue samples were weighed, mixed with an equal volume of pure water, and homogenized using a cryogenic grinder. The supernatant was obtained by centrifuging at 800 × g for 10 minutes, and the cuprous ion content was measured using a copper detection kit (E010-1–1, Nanjing, China).

### 5.10 Dual-luciferase reporter assay

Target gene promoter sequences were cloned into a vector with a luciferase reporter gene and transfected into HEK293T cells. After 24 hours, cells were lysed with a dual luciferase assay kit (Promega) following the manufacturer’s protocol to measure firefly and Renilla luciferase activities. Relative promoter activity was determined by the firefly to Renilla luciferase activity ratio.

### 5.11 Fluorescence in situ hybridization (FISH) and immunofluorescence (IF)

The 3’-FAM fluorescently labeled tRF-Gly-M3 and 3’-Cy3 fluorescently labeled glutaminase (GLS) were synthesized by GenePharma (Shanghai, China). The sequence is shown in S1 Table. The distribution of tRF-Gly-M3 in ovarian tissues and its co-localization with GLS were examined using a FISH kit from the same supplier. Fixed cell coverslips or frozen tissue sections were permeabilized with 0.1% Triton X-100 for 15 minutes, followed by overnight incubation with the probe solution at 37°C. Nuclei were stained with DAPI and visualized using a confocal microscope.

### 5.12 Western blot analysis

Cells were lysed using RIPA buffer with 1% PMSF (ST2573, Beyotime Bio) or NP40 lysis buffer, followed by centrifugation at 12,000 × g for 20 minutes. Soluble proteins in the supernatant and insoluble proteins in the pellet were collected separately. The insoluble fraction was then subjected to additional sonication. Appropriate volumes of 5 × SDS loading buffer were added to each sample, which were then denatured at 95°C for 5–10 minutes. The proteins were then boiled and resolved via 4%–12% SDS-PAGE. Membranes were blocked with 5% skimmed milk for 1 hour at room temperature, incubated with primary antibodies overnight at 4°C, and then with secondary antibodies for 1 hour at room temperature. Protein bands were detected using ECL and analyzed for grayscale values using ImageJ software. The antibodies are shown in S2 Table.

### 5.13 Quantitative reverse transcription PCR (qRT-PCR)

Total RNA was extracted with TRIzol reagent, followed by reverse transcription of small RNA and mRNA using the Reverse Transcription Kit (Takara Bio) as per the manufacturer’s protocol. qPCR amplification was conducted with SYBR Green Master Mix (Takara Bio) at 95°C for 15 s and 60°C for 30 s. Primers were designed via Prime 6.0, with U6, GAPDH, or β-actin serving as internal reference genes. Relative expression of target genes was calculated using the 2^-ΔΔCt^ method. Primer sequences are detailed in S3 Table.

### 5.14 Mitochondrial enzyme and metabolite analysis

Cuproptosis inducer-treated cells were trypsinized, washed twice with PBS, transferred to 1.5 mL EP tubes, and lysed using the lysis solution from the respective kit as per instructions. The following kits were employed: α-ketoglutarate content assay kit (AKAC016U, Beijing Boxbio), malic acid (L-MA) content kit (G0862W48, Geruisi Bio), glutaminase (GLS) kit (G0407W, Geruisi Bio), glutamate (Glu) content assay kit (AKAM002C, Shanghai Sangon Bio), lactate assay kit (A019-2–1, Nanjing JianCheng Bio), LDH activity assay kit (A020-1–2, Nanjing JianCheng Bio), SDH enzyme content assay kit (G0856W, Geruisi Bio), glucose (Glu) test kit (F006-1–1, Nanjing JianCheng Bio), and ATP detection kit (S0026-2, Beyotime Bio).

### 5.15 Mitochondrial activity and oxidative stress analysis

Mitochondrial Morphology and Structure: After treatment, cells were harvested at 700 × g for 5 minutes, and fixed with electron microscope fixative. Water was removed using ethanol, and the samples were resin-embedded, sectioned, stained, and examined via Transmission Electron Microscopy to assess mitochondrial number, size, and shape. Mito-Tracker Red fluorescence staining was employed to evaluate mitochondrial morphological integrity and activity. In vitro cultured oocytes were treated with a mitotracker probe solution (final concentration 50 nM), incubated at 37°C for 30 minutes, and then observed and photographed using a fluorescence inverted microscope (Leica, Germany).

ROS measurement: After treatment, cytochrome ROS levels in oocytes were assessed using the ROS-specific fluorescent probe DCFH-DA (HY-D094, MedChemExpress). Samples were incubated at 37°C for 30 minutes, then observed and imaged with a fluorescence inverted microscope.

### 5.16 Oxygen consumption rates (OCR) and extracellular acidification rates (ECAR)

Mitochondrial function was assessed using the Seahorse XFe24 Extracellular Flux Analyzer. Cells were seeded at a density of 4 × 10^4^ per well in Seahorse XF24 cell culture plates and incubated for 24 hours prior to transfection with tRF mimics or mimic-nc. Upon reaching 80% confluency, the culture medium was replaced with Seahorse XF medium. Sequentially, 0.5 μM oligomycin, 0.5 μM FCCP, and 1 μM antimycin A were added to the plates. Basal respiration, maximal respiration, and ATP production parameters were then measured. Data analysis was conducted using Seahorse Wave software, with all OCR and ECAR measurements normalized to protein content on a per-well basis.

### 5.17 Statistical analysis

Data were presented as mean ± standard deviation (SD). Statistical analyses were conducted using GraphPad Prism 9.0 (GraphPad Software, USA). Significance was assessed via two-tailed Student’s t-test or one-way ANOVA with Bonferroni post hoc test for multiple comparisons. A *p*-value of less than 0.05 was considered significant. Significance levels are indicated as *, **, ***, and ****, representing *p*-values of <0.05, < 0.01, < 0.001, and <0.0001, respectively.

## Supporting information

S1 Fig Tissue targeting of copper overload in mice. (A) Three-dimensional imaging of monovalent copper in mice after tail vein injection of copper ion probes at various intervals; (B) AUC (area under curve) statistics from metabolic cage analysis in mice; (C-D) Three-dimensional fluorescence imaging of ovary and uterus, with quantification of monovalent copper fluorescence intensity (C) and organ images (D) in mice; (E) Quantification of copper probe fluorescence intensity in mice over time; (F) Three-dimensional fluorescence imaging of primary copper-metabolizing organs in mice, monovalent copper (Coppersensor, red) and bivalent copper (Rhodamine B hydrazide, green). All *p* values were calculated using two-tailed unpaired Student’s t test; *p < 0.05, **p < 0.01, ***p < 0.001.
(TIF)

S2 Fig Copper accumulation in mouse granulosa cells. (A) Rhodamine B hydrazide (RBH) assay for Cu^2+^ detection; (B) Copper ion content analysis; (C) Fluorescence intensity statistics for copper ions; (D) Coppersensor-1 staining for Cu⁺ accumulation; (E) Schematic depiction of the primary functions of the two mitochondrial types in follicular granulosa cells; (F) Transmission electron microscopy images of granulosa cell mitochondria, lamellar cristae (blue) and tubular cristae (purple). All *p* values were calculated using two-tailed unpaired Student’s t test; *p < 0.05, **p < 0.01, ***p < 0.001.
(TIF)

S3 Fig The generation of tiRNA-Gly-M3 from ANG-spliced tRNA modulates gene expression through Ago proteins. (A) RT-qPCR analysis determines four tRFs with high expression in tsRNA-seq after cuproptosis treatment, and predicted binding maps of tRF-Gly-M3’s seed sequence to the CDS region of GLS; (B-C) Western blot assays quantified alterations in the expression of key cuproptosis proteins after transfection with tRF-Gly-M3 mimics and inhibitors; (D-F) Ago knockdown efficiency assay; (G-I) RT-qPCR analyses the expression of GLS mRNA after co-treatment of Ago knockdown and tiRNA-Gly-M3 mimics; (J) CCK-8 assay to evaluate granulocyte activity inhibition post-transfection with si-ANG; (K) RT-qPCR to assess transfection efficiency of the three ANG-interfering strands; (L) RT-qPCR for tiRNA-Gly-M3 after si-ANG transfection. (M) Visualise the molecular-docking results using PyMOL. (N-O) Perform native RIP-qPCR with an anti-AGO3 antibody to assess binding between tiRNA-Gly-M3 and AGO3; IgG as the control. The data in (A)–(L) represents the means ± SD of ≥3 independent experiments. All *p* values were calculated using two-tailed unpaired Student’s t test; *p < 0.05, **p < 0.01, ***p < 0.001.
(TIF)

S4 Fig Graphical abstract. Created in BioRender. Wu, S. (2026) https://BioRender.com/9pbypto (TIF)

S1 Table Sequences of siRNA, inhibitor, mimic and probe used in this study. (XLSX)

S2 Table Antibodies involved in this study. (XLSX)

S3 Table Sequence of real-time PCR primers. (XLSX)

S1 Data Data. (XLSX)

S1 File File. (DOCX)
