# Supplementary figures and images for: A novel role of tRNA-derived fragments in porcine granulosa-oocyte cell communication and cuproptosis
Source: PLoS Genet. 2026 Apr 30;22(4):e1012119. doi: 10.1371/journal.pgen.1012119 (PMC13132174; doi:10.1371/journal.pgen.1012119)

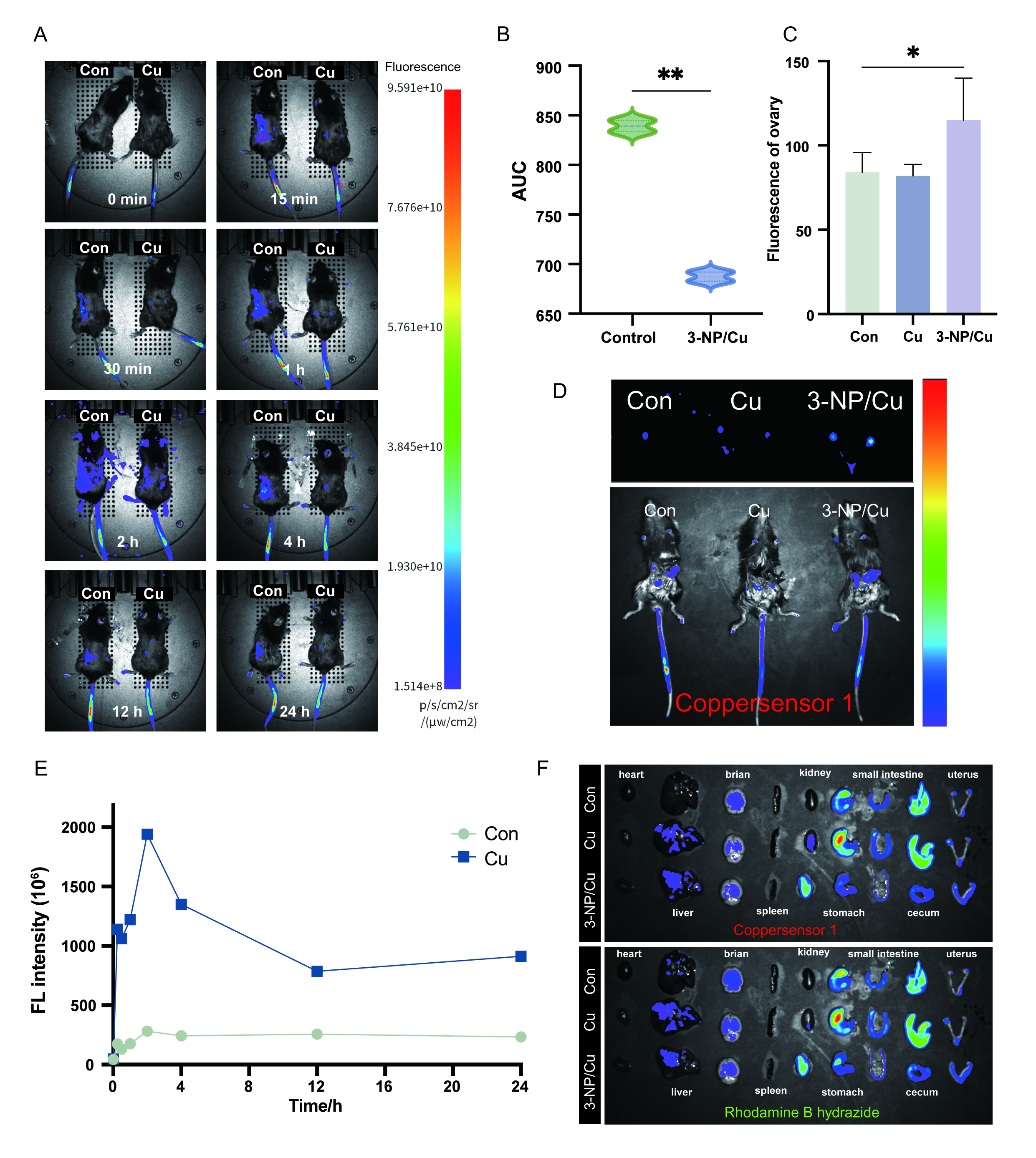

Supplement: S1 Fig — (A) Three-dimensional imaging of monovalent copper in mice after tail vein injection of copper ion probes at various intervals; (B) AUC (area under curve) statistics from metabolic cage analysis in mice; (C-D) Three-dimensional fluorescence imaging of ovary and uterus, with quantification of monovalent copper fluorescence intensity (C) and organ images (D) in mice; (E) Quantification of copper probe fluorescence intensity in mice over time; (F) Three-dimensional fluorescence imaging of primary copper-metabolizing organs in mice, monovalent copper (Coppersensor, red) and bivalent copper (Rhodamine B hydrazide, green). All p values were calculated using two-tailed unpaired Student’s t test; *p < 0.05, **p < 0.01, ***p < 0.001. (TIF) [file pgen.1012119.s001.tif]

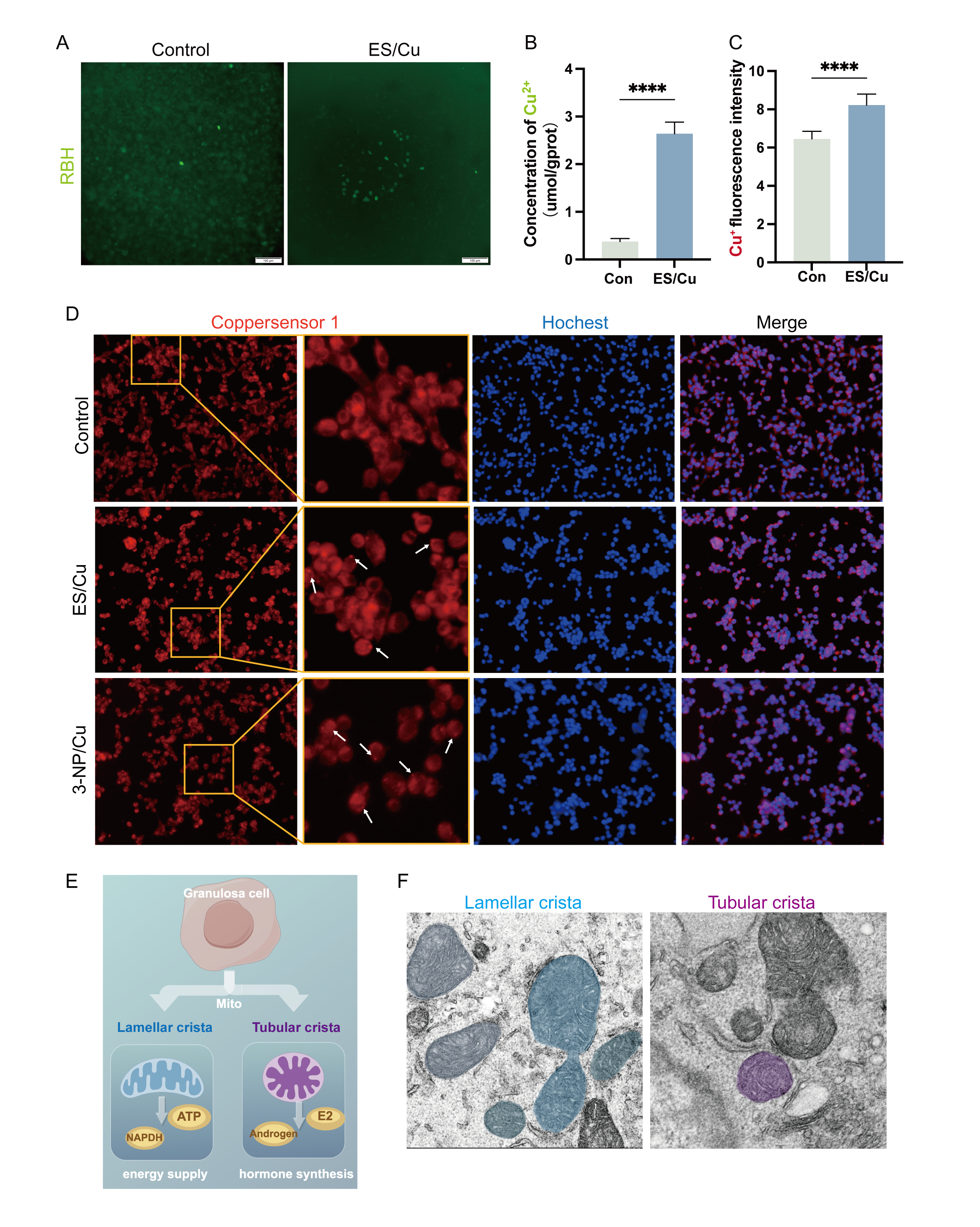

Supplement: S2 Fig — (A) Rhodamine B hydrazide (RBH) assay for Cu2+ detection; (B) Copper ion content analysis; (C) Fluorescence intensity statistics for copper ions; (D) Coppersensor-1 staining for Cu⁺ accumulation; (E) Schematic depiction of the primary functions of the two mitochondrial types in follicular granulosa cells; (F) Transmission electron microscopy images of granulosa cell mitochondria, lamellar cristae (blue) and tubular cristae (purple). All p values were calculated using two-tailed unpaired Student’s t test; *p < 0.05, **p < 0.01, ***p < 0.001. (TIF) [file pgen.1012119.s002.tif]

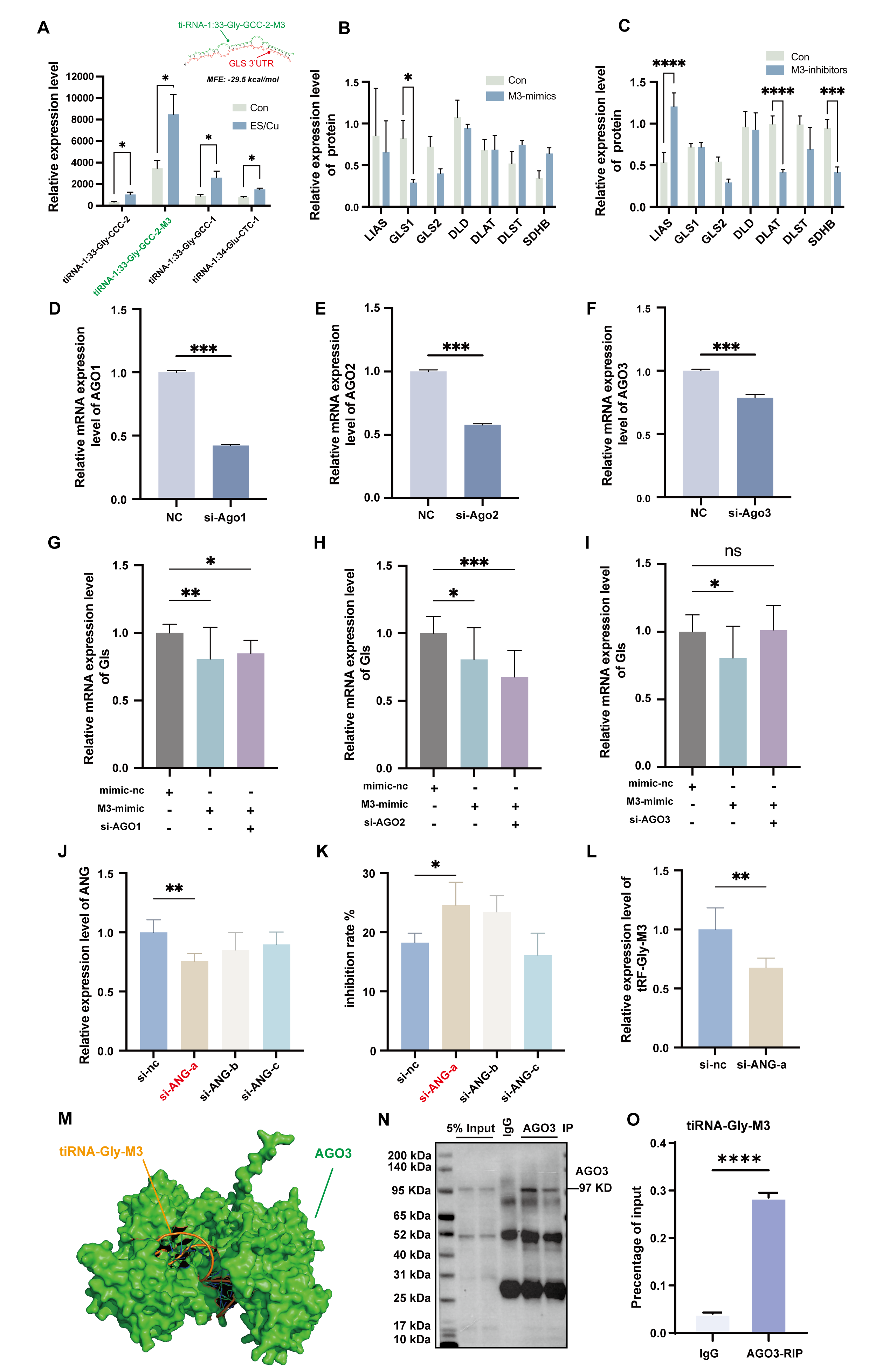

Supplement: S3 Fig — (A) RT-qPCR analysis determines four tRFs with high expression in tsRNA-seq after cuproptosis treatment, and predicted binding maps of tRF-Gly-M3’s seed sequence to the CDS region of GLS; (B-C) Western blot assays quantified alterations in the expression of key cuproptosis proteins after transfection with tRF-Gly-M3 mimics and inhibitors; (D-F) Ago knockdown efficiency assay; (G-I) RT-qPCR analyses the expression of GLS mRNA after co-treatment of Ago knockdown and tiRNA-Gly-M3 mimics; (J) CCK-8 assay to evaluate granulocyte activity inhibition post-transfection with si-ANG; (K) RT-qPCR to assess transfection efficiency of the three ANG-interfering strands; (L) RT-qPCR for tiRNA-Gly-M3 after si-ANG transfection. (M) Visualise the molecular-docking results using PyMOL. (N-O) Perform native RIP-qPCR with an anti-AGO3 antibody to assess binding between tiRNA-Gly-M3 and AGO3; IgG as the control. The data in (A)–(L) represents the means ± SD of ≥3 independent experiments. All p values were calculated using two-tailed unpaired Student’s t test; *p < 0.05, **p < 0.01, ***p < 0.001. (TIF) [file pgen.1012119.s003.tif]

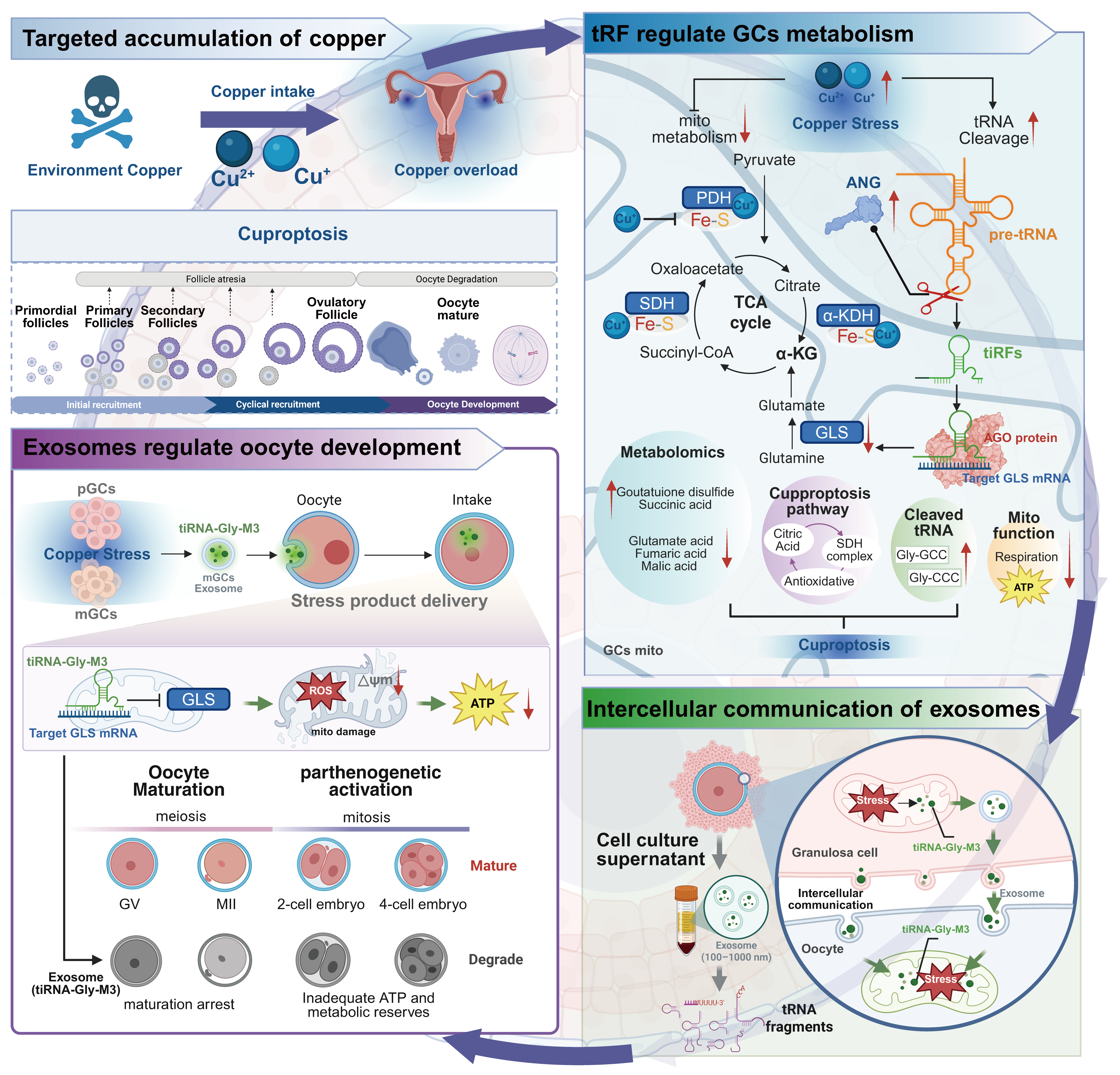

Supplement: S4 Fig — (TIF) [file pgen.1012119.s004.tif]
