# Supplementary material for: A novel role of tRNA-derived fragments in porcine granulosa-oocyte cell communication and cuproptosis
Source: PLoS Genet. 2026 Apr 30;22(4):e1012119. doi: 10.1371/journal.pgen.1012119 (PMC13132174; doi:10.1371/journal.pgen.1012119)

**Figure.3**

**Fig.3-D**

**FDX1**

**
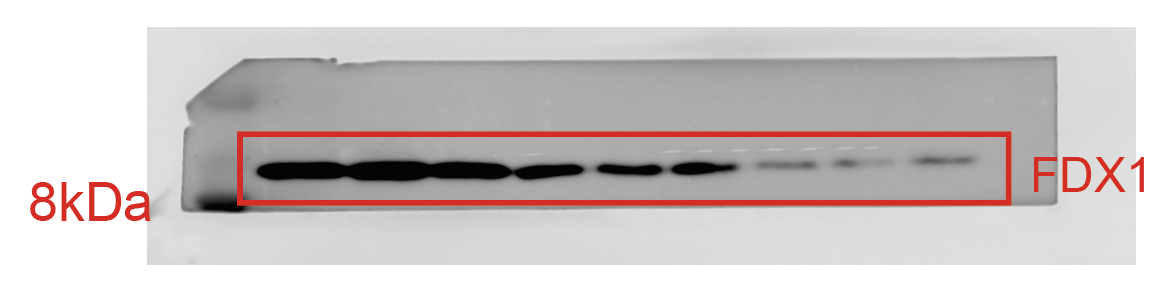
**

**SDHB**

**
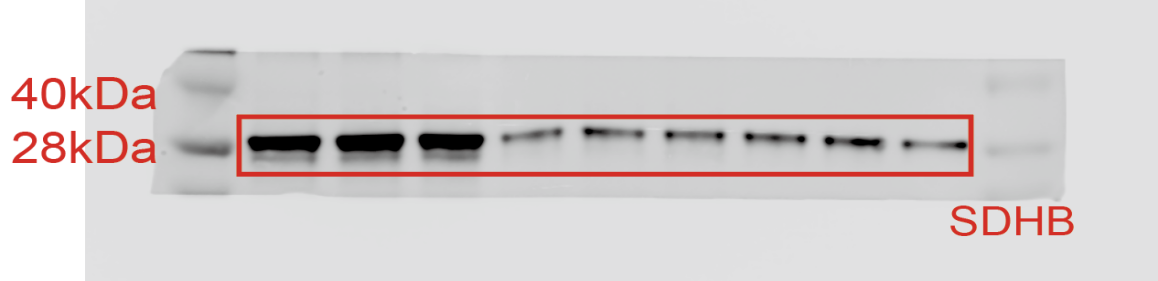
**

**POLD1**

**
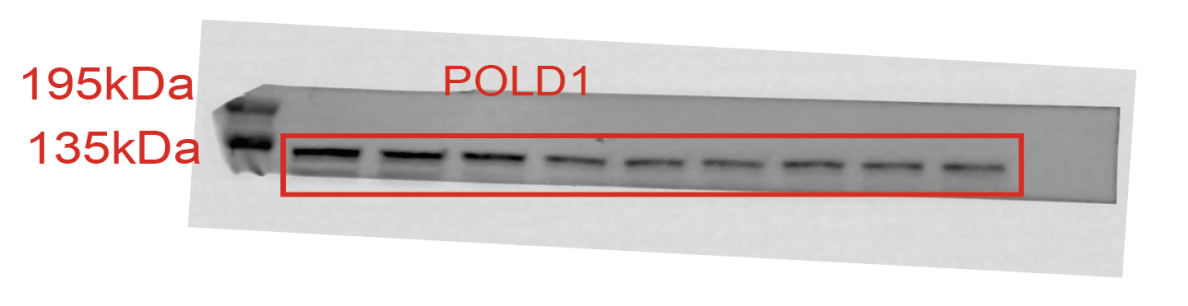
**

**PDHA1**

**
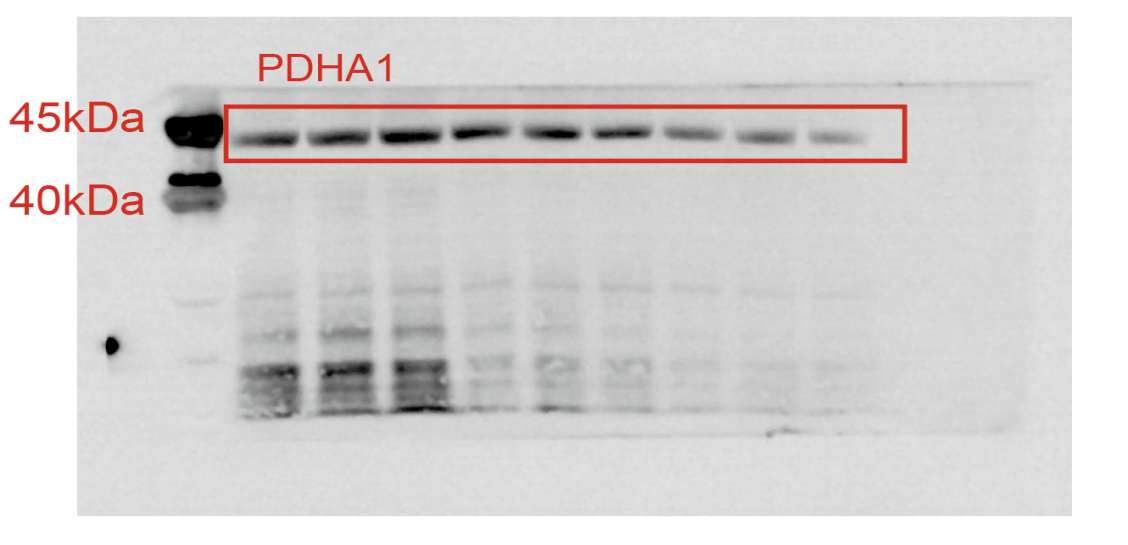
**

**PDHB**

**
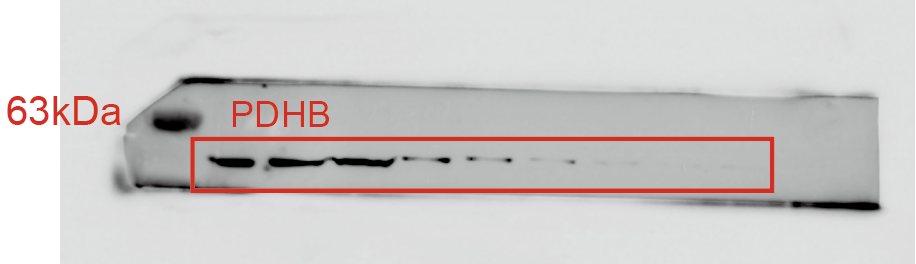
**

**DLAT**

**
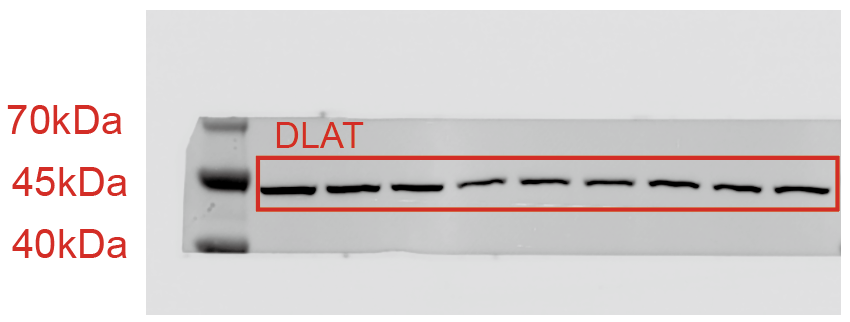
**

**DLD**

**
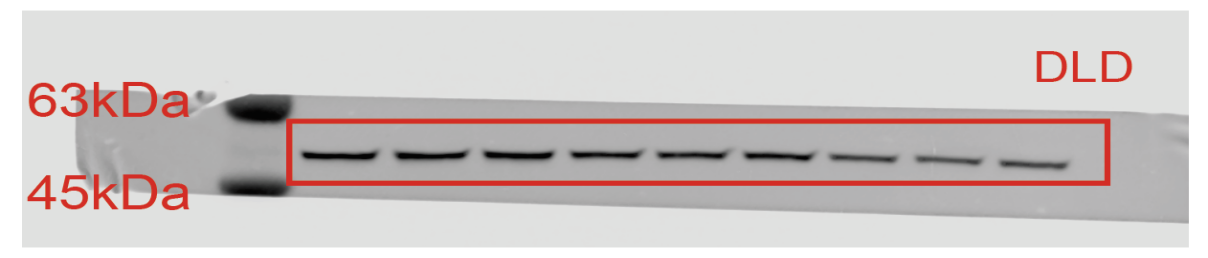
**

**DLST**

**
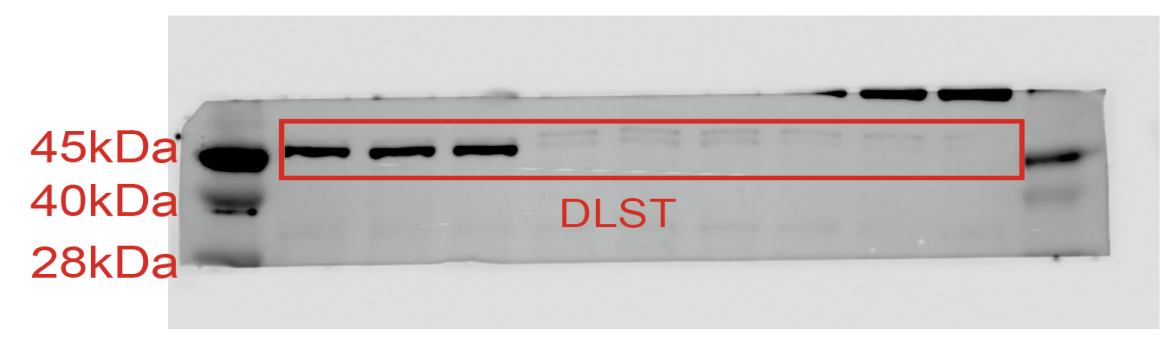
**

**GLS**

**
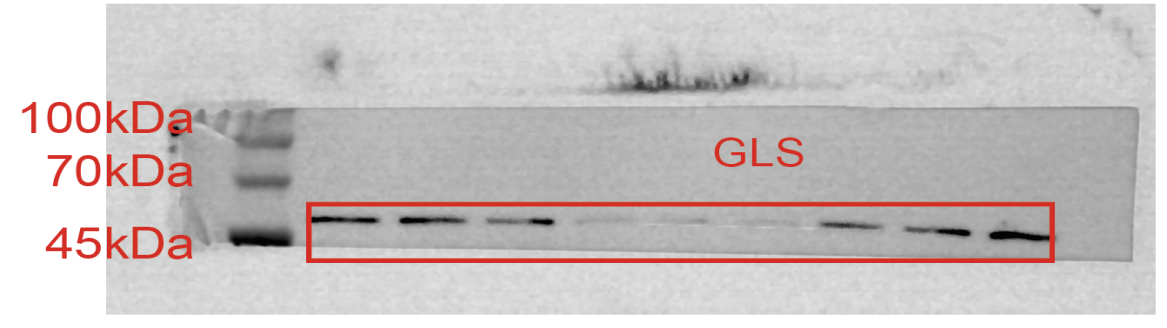
**

**β-tubulin**

**
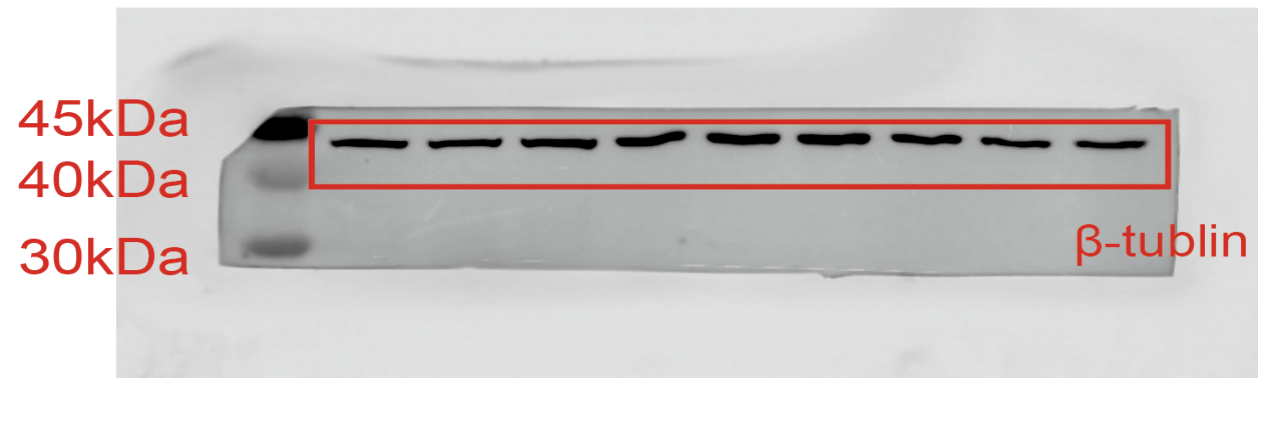
**

**Figure.6**

**Fig.6-C**

**LIAS**

**
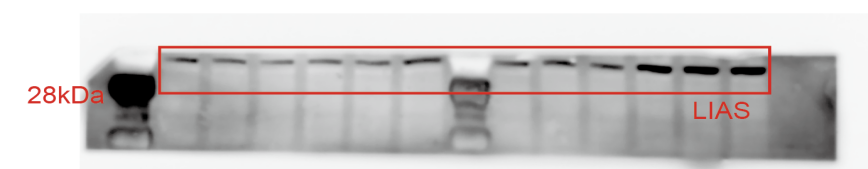
**

**GLS**

**
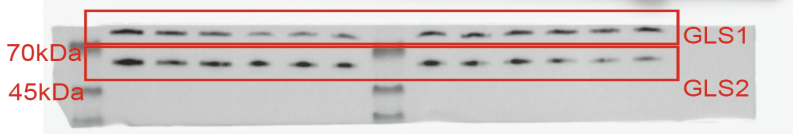
**

**DLD**

**
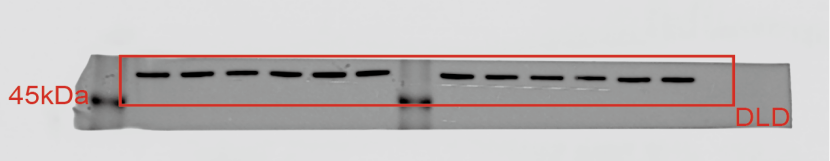
**

**DLAT**

**
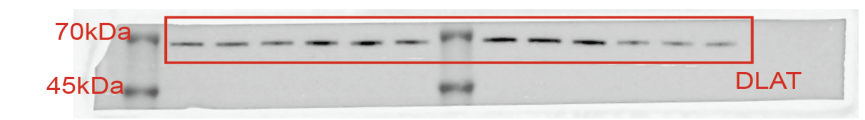
**

**DLST**

**
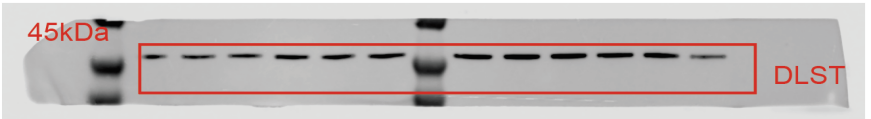
**

**SDHB**

**
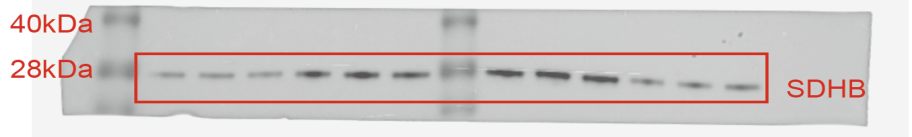
**

**β-actin**

**
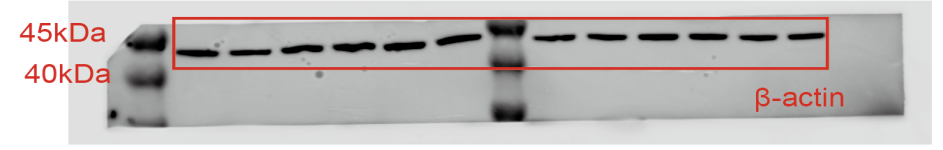
**

**Fig.6-H**

**GLS**

**
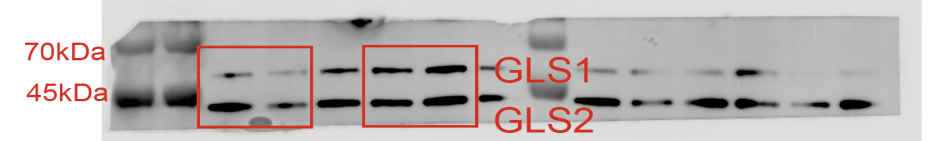
**

**β-actin**

**
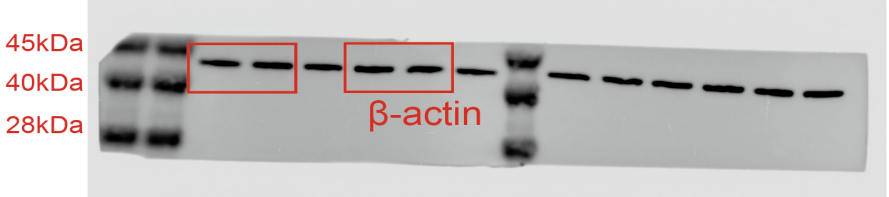
**

**Figure.8**

**Fig.8-C**

**TSG101**

**
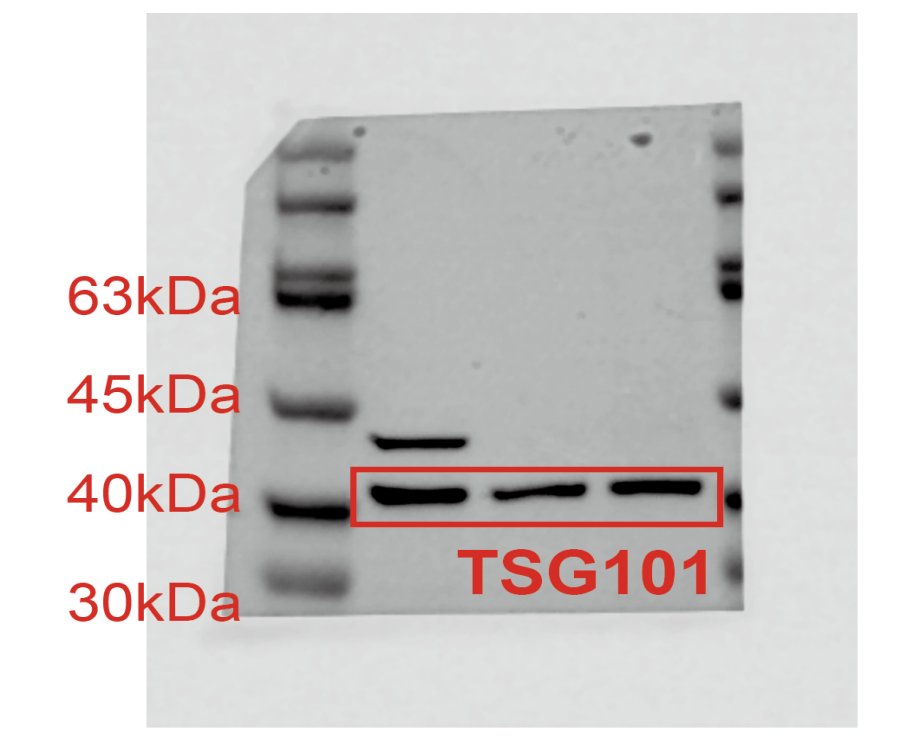
**

**HSP70**

**
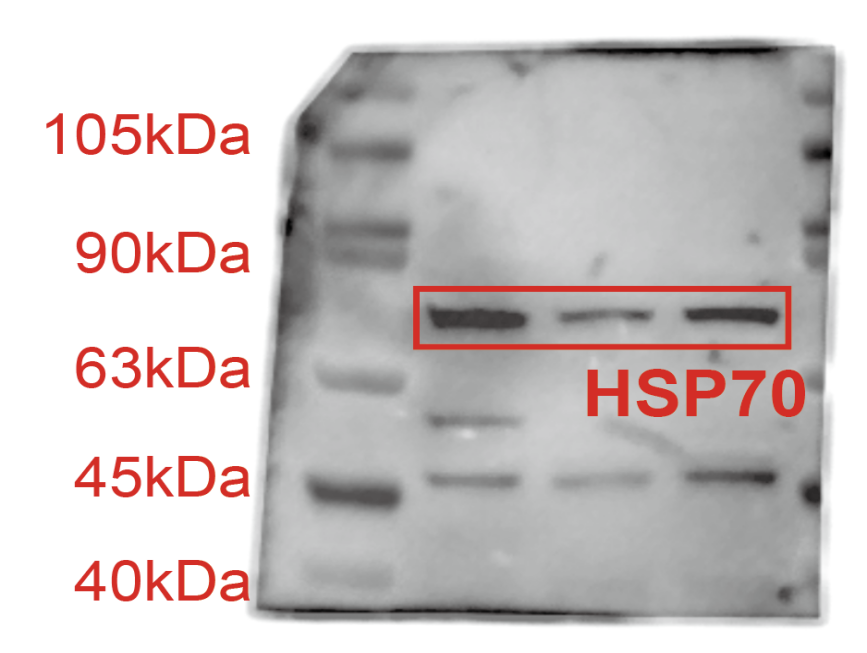
**

**CD81**

**
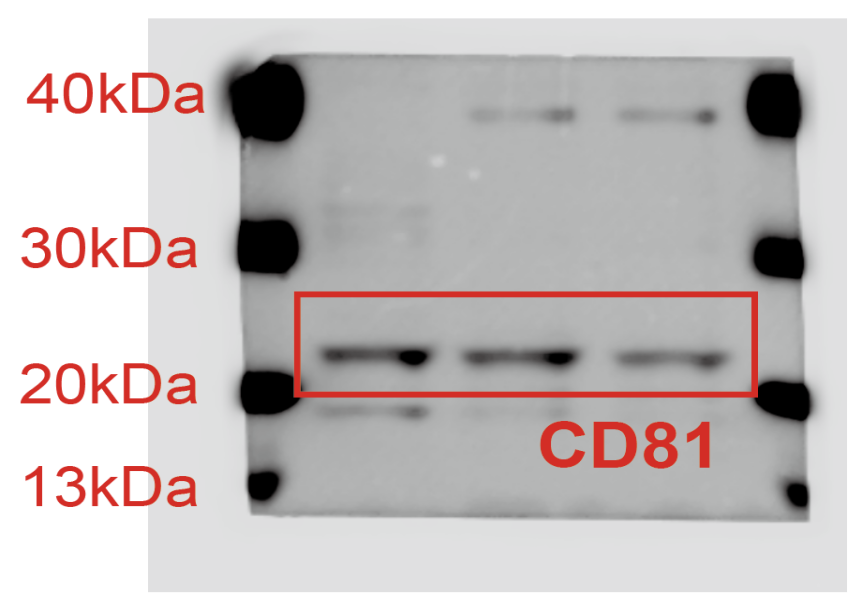
**

**CD9**

**
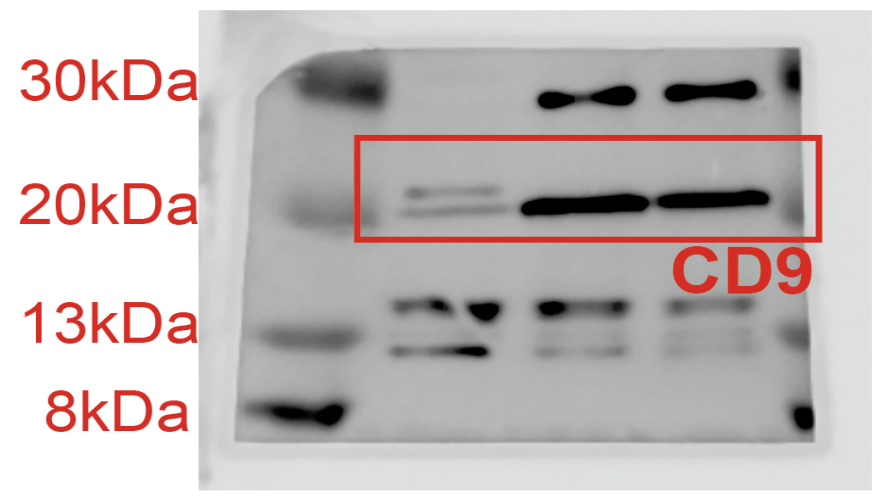
**

**Caln**

**
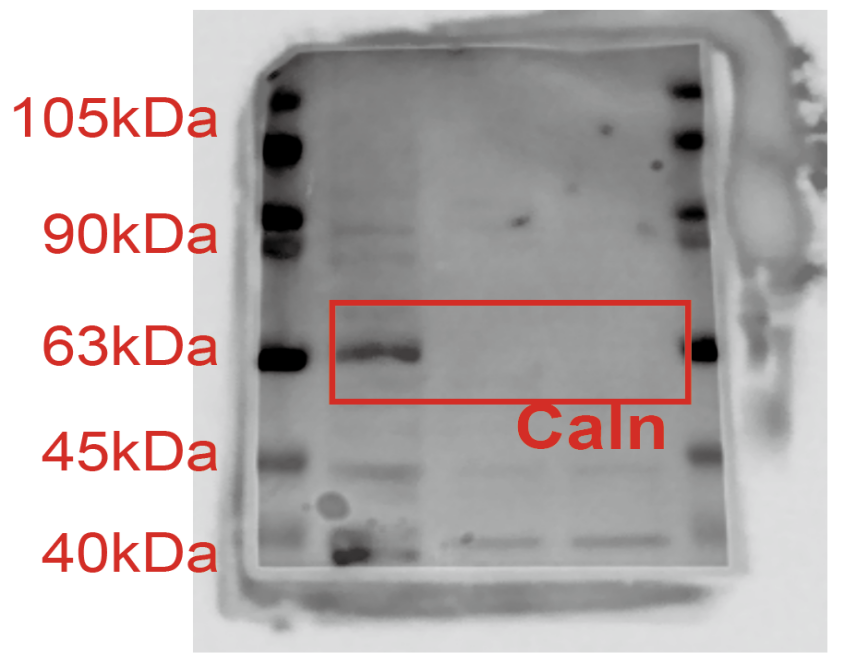
**

**Fig.8-G**

**Ovary-U6**

**
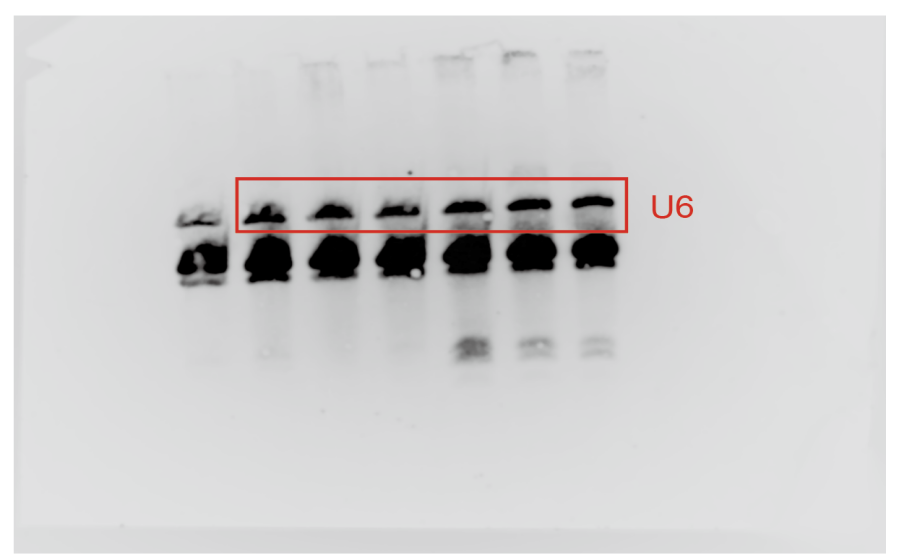
**

**Ovary-tiRNA-Gly-M3**

**
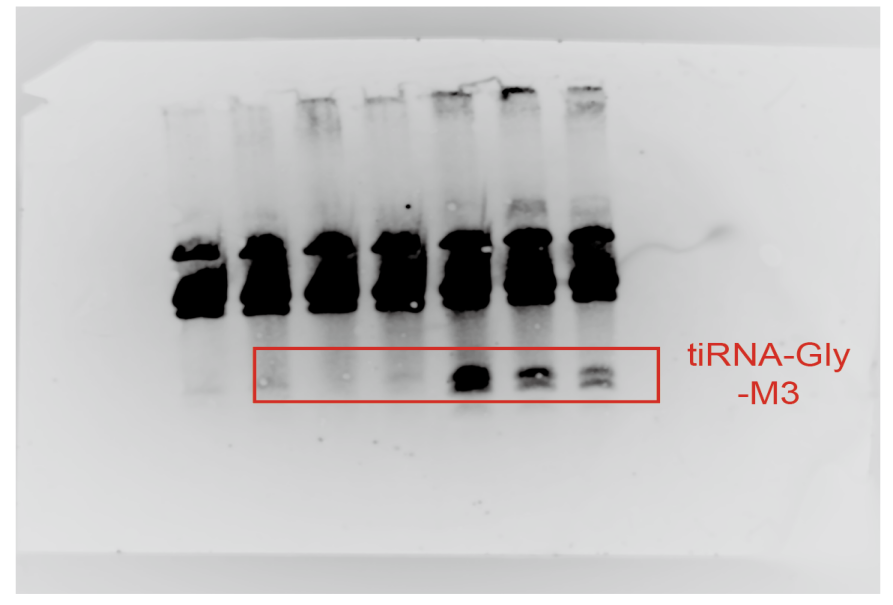
**

**Granulosa cell-U6**

**
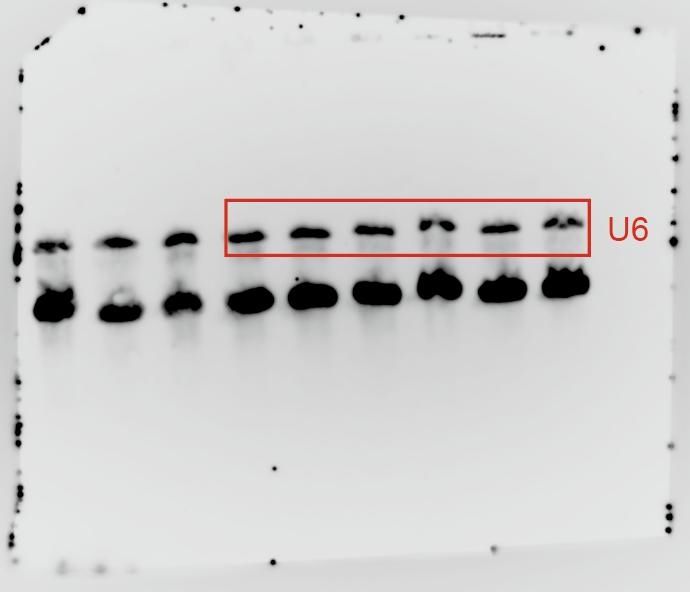
**

**Ovary-tiRNA-Gly-M3**

**
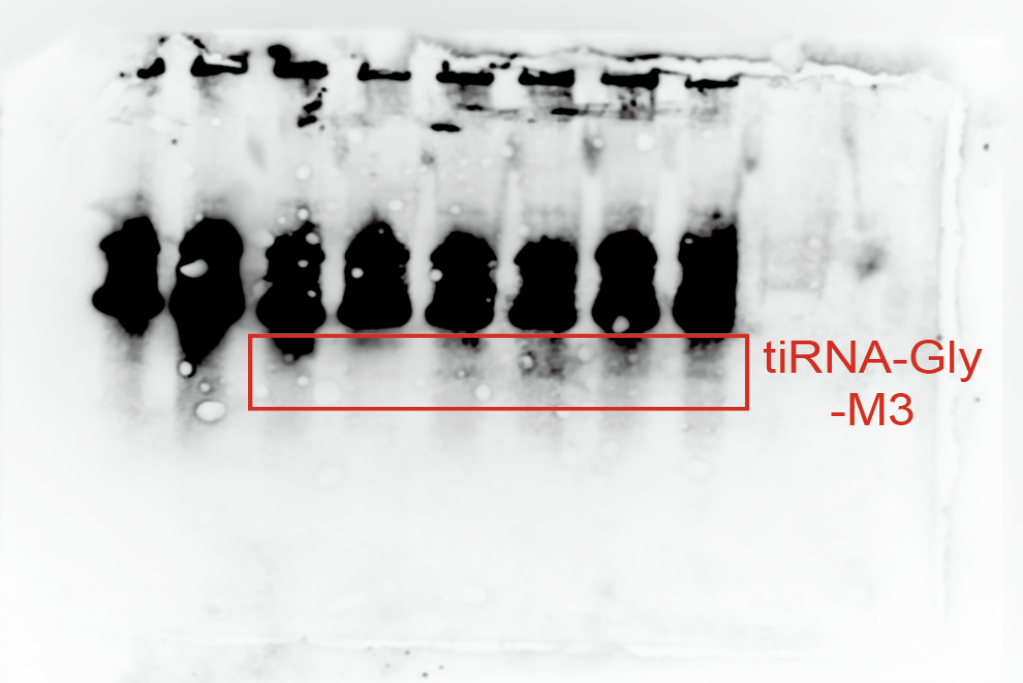
**

**Fig.3-N**

**AGO3-RIP**


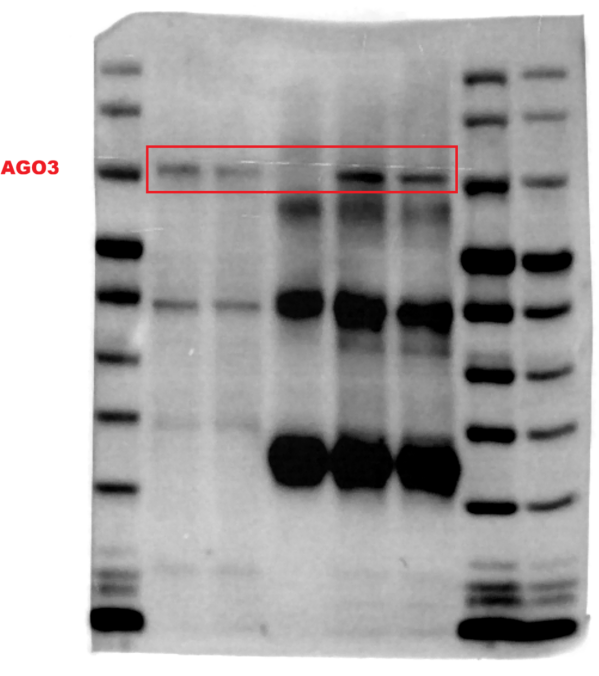

Supplement: S1 File — (DOCX) [file pgen.1012119.s009.docx]
